# Utilization of ferulic acid in *Aspergillus niger* requires the transcription factor FarA and a newly identified Far-like protein (FarD) that lacks the canonical Zn(II)_2_Cys_6_ domain

**DOI:** 10.3389/ffunb.2022.978845

**Published:** 2022-11-08

**Authors:** Mark Arentshorst, Jos Reijngoud, Daan J. C. van Tol, Ian D. Reid, Yvonne Arendsen, Herman J. Pel, Noël N. M. E. van Peij, Jaap Visser, Peter J. Punt, Adrian Tsang, Arthur F. J. Ram

**Affiliations:** ^1^ Microbial Sciences, Institute of Biology Leiden, Leiden University, Leiden, Netherlands; ^2^ Centre for Structural and Functional Genomics, Concordia University, Montreal, QC, Canada; ^3^ DSM Biosciences and Process Innovation, Center for Biotech Innovation, Delft, Netherlands; ^4^ DSM Food and Beverage, Center for Food Innovation, Delft, Netherlands; ^5^ Fungal Genetics and Technology Consultancy, Wageningen, AJ, Netherlands

**Keywords:** transcriptional regulation, peroxisome, hydroxycinnamic acids, feruloyl esterase, fatty acids, CoA-dependent β-oxidative pathway

## Abstract

The feruloyl esterase B gene (*faeB*) is specifically induced by hydroxycinnamic acids (e.g. ferulic acid, caffeic acid and coumaric acid) but the transcriptional regulation network involved in *faeB* induction and ferulic acid metabolism has only been partially addressed. To identify transcription factors involved in ferulic acid metabolism we constructed and screened a transcription factor knockout library of 239 *Aspergillus niger* strains for mutants unable to utilize ferulic acid as a carbon source. The *ΔfarA* transcription factor mutant, already known to be involved in fatty acid metabolism, could not utilize ferulic acid and other hydroxycinnamic acids. In addition to screening the transcription factor mutant collection, a forward genetic screen was performed to isolate mutants unable to express *faeB.* For this screen a *PfaeB-amdS* and *PfaeB-lux_613_* dual reporter strain was engineered. The rationale of the screen is that in this reporter strain ferulic acid induces *amdS* (acetamidase) expression *via* the *faeB* promoter resulting in lethality on fluoro-acetamide. Conidia of this reporter strain were UV-mutagenized and plated on fluoro-acetamide medium in the presence of ferulic acid. Mutants unable to induce *faeB* are expected to be fluoro-acetamide resistant and can be positively selected for. Using this screen, six fluoro-acetamide resistant mutants were obtained and phenotypically characterized. Three mutants had a phenotype identical to the *farA* mutant and sequencing the *farA* gene in these mutants indeed showed mutations in FarA which resulted in inability to growth on ferulic acid as well as on short and long chain fatty acids. The growth phenotype of the other three mutants was similar to the *farA* mutants in terms of the inability to grow on ferulic acid, but these mutants grew normally on short and long chain fatty acids. The genomes of these three mutants were sequenced and allelic mutations in one particular gene (NRRL3_09145) were found. The protein encoded by NRRL3_09145 shows similarity to the FarA and FarB transcription factors. However, whereas FarA and FarB contain both the Zn(II)_2_Cys_6_ domain and a fungal-specific transcription factor domain, the protein encoded by NRRL3_09145 (FarD) lacks the canonical Zn(II)_2_Cys_6_ domain and possesses only the fungal specific transcription factor domain.

## Introduction

Plant biomass represents an exploitable renewable carbon source from which to generate both fuels and chemicals. However, its robust nature generally prevents it from being utilized directly (Oliveira et al., 2015; Fatma et al., 2018). Extensive pretreatment of raw plant biomass is a general requirement for its exploitation, driving up the costs of the chemical exploitation process (Mosier et al., 2005; Carroll and Somerville, 2009; Sun et al., 2022). An alternative approach is to use enzymes to facilitate exploitation of raw biomass. Fungi are especially well equipped to deal with the robustness of plant biomass, being able to efficiently degrade plant cell walls through the secretion of cell wall degrading enzymes. Using complex regulatory responses, fungi are able to selectively secrete enzymes to utilize the nutrients available in their environment (Glass et al., 2013; Guerriero et al., 2015; Benocci et al., 2017; Niu et al., 2017). This property makes fungi ideal organisms for industrial exploitation of plant biomass. However, to optimize the exploitation process, the complex regulatory network regulating the expression of enzymes involved in the degradation process has to be understood thoroughly (see for reviews Huberman et al., 2016; Benocci et al., 2017; Alazi and Ram, 2018; Wu et al., 2020).

An important factor in acquiring robustness of plant biomass against fungal enzymatic degradation is the presence of lignin and other aromatic compounds including tannins and hydroxycinnamic acids such as ferulic acid. The presence of these aromatic compounds and the formation of covalent lignin-hydroxycinnamate-hemicellulose complexes together with the toxicity of these compounds prevent easy degradation by hemicellulolytic enzymes (Tabka et al., 2006; Selig et al., 2008; Wong et al., 2013). In this study, we have focused on the regulation of enzymes involved in the removal of ferulic acid from the plant cell wall and the subsequent metabolism of ferulic acid. Plants use ferulic acid to establish ester linkages between, for instance, arabinoxylans and pectins (Saulnier and Thibault, 1999; Mäkelä et al., 2015; Hatfield et al., 2017). Fungal degradation of ferulic acid from plant cell wall biomass is facilitated by ferulyol esterases. Several ferulyol esterases (FaeA-D) have been identified in *A. niger*, with expression of the genes being induced only under specific conditions. FaeA expression is induced on xylose, arabinose and several aromatics, whereas FaeB expression is only induced on aromatics (de Vries et al., 2002; Reijngoud et al., 2021). FaeC is generally lowly expressed (Dilokpimol et al., 2017; Oliveira et al., 2019; Lubbers et al., 2020) and no data are currently available for FaeD. The feruloyl esterases of *A. niger* display different substrate preferences and varying abilities to release ferulic acid or di-ferulic acids from plant biomass. FaeA is most active towards wheat arabinoxylan, whereas FaeB shows higher activity towards sugar beet pectin over arabinoxylan (de Vries et al., 2002). A more recent study indicated that FaeC shares the preference of FaeA to hydrolyse ferulic acid from xylan over pectin (Dilokpimol et al., 2017).

**Figure 1:**
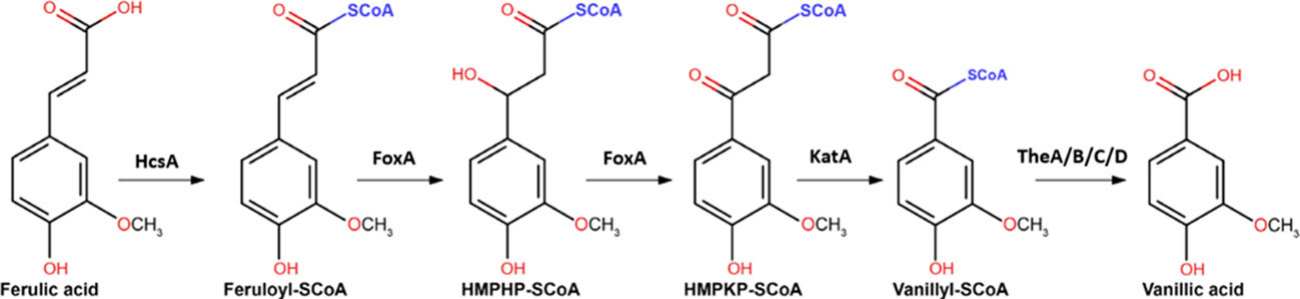
The proposed CoA-dependent β-oxidative pathway for the metabolism of ferulic acid to vanillic acid. Genes encoding the proposed enzymes include HcsA (NRRL3_05989; putative hydroxycinnamate-CoA synthase), FoxA (NRRL3_00672; putative cinnamoyl-CoA hydratase/dehydrogenase) and KatA (NRRL3_05990; putative 3-ketoacyl CoA thiolase), and TheA-TheD (NRRL3_00621, NRRL3_01539, NRRL3_02735 and NRRL3_06009) are indicated (Figure adapted from Lubbers et al., 2021a).

After the release of ferulic acid from the plant cell wall biomass, for further metabolism ferulic acid has to enter the cell *via* ferulic acid transporters. Moreover, it has recently been established that metabolism of ferulic acid involves genes from the peroxisomal CoA-dependent β-oxidative pathway (Lubbers et al., 2021a). Although in fungi the CoA-dependent β-oxidative pathway was mainly known for the degradation of fatty acids, plants have been shown previously to utilize the pathway for hydroxycinnamic acid degradation (Widhalm and Dudareva, 2015). Candidate genes involved in ferulic acid metabolism were identified in a comparative transcriptome analysis in combination with *in silico* functional predictions of strongly induced genes. These genes were subsequently deleted and the ability of the mutants to grow on different aromatics was analysed (Lubbers et al., 2021a). This led to the identification of HcsA (NRRL3_05989; putative hydroxycinnamate-CoA synthase), FoxA (NRRL3_00672; putative cinnamoyl-CoA hydratase/dehydrogenase) and KatA (NRRL3_05990; putative 3-ketoacyl CoA thiolase) that are required for growth on ferulic acid. The final step of the CoA-dependent oxidative pathway is proposed to be catalyzed by a thioesterase, with four thioesterases (TheA-D; NRRL3_00621, NRRL3_01539, NRRL3_02735 and NRRL3_06009) identified in the fungal genome. Individual disruption of these genes did not result in reduced growth on ferulic acid, suggesting that there is functional redundancy in this step of the pathway in *A. niger* (Lubbers et al., 2021a) (**Figure 1**). Subsequent genes involved in vanillic acid metabolism have been recently identified as well (Lubbers et al., 2021b).

Both the genes encoding the enzymes involved in ferulic acid metabolism and the gene encoding feruloyl esterase B (FaeB) are induced in the presence of ferulic acid and other hydroxycinnamic acids (de Vries et al., 2002; Reijngoud et al., 2021; Lubbers et al., 2021a), suggesting that they might be controlled by the same transcription factor(s). We previously performed a screen to isolate *A. niger* mutants with constitutive expression of the *faeB* gene by using *PfaeB-amdS* and *PfaeB-luciferase* reporter strains (Reijngoud et al., 2021). More than 100 of these mutants were isolated and all were found to be mutated in the *creA* gene encoding the major player in carbon catabolite repression. The results from that study showed that loss of function of CreA resulted in constitutive expression of *faeB*. Unfortunately, this study did not yield other mutants in which a gain of function was introduced in the proposed transcriptional activator involved in the induction of *faeB*. Using the same screening principle but using other reporter strains, constitutive mutants in transcription factor involved in the induction of pectinases (GaaR), arabinases (AraR) and xylanases (XlnR) have been identified, showing the potential of this approach (Alazi et al., 2019; Reijngoud et al., 2019, Reijngoud and Ram, unpublished).

In this study, the search for transcription factors involved in *faeB* induction and ferulic acid metabolism was continued by using the same *PfaeB-amdS* and *PfaeB-luciferase* reporter strains (Reijngoud et al., 2021) but now using a new *amdS* counter selection strategy, and by constructing and screening a large collection of *A. niger* transcription factor deletion mutants. In the genome of *A. niger* approximately 660 predicted transcription factors, including approximately 440 transcription factors with a Zn(II)_2_Cys_6_ domain, are present. From the 660 genes encoding predicted transcription factors, 280 were selected for targeted deletion. These complementary approaches resulted in the identification of the FarA Zn(II)_2_Cys_6_ transcription factor as a transcription factor required not only for the metabolism of fatty acids, but also for the metabolism of hydroxycinnamic acids, including ferulic acid. The forward genetic screen identified, in addition to *farA*, a gene encoding a very peculiar protein (FarD) which is a new member of the Far-like transcription factor family. In contrast to FarA and FarB, FarD lacks the canonical Zn(II)_2_Cys_6_ domain.

## Material and methods

### Strains, media and growth conditions

The *A. niger* strains used in this study are listed in **Table 1**. A*. niger* strain JR11.1 contains two reporter genes (*PfaeB-amdS* and *PfaeB-lux_613_*_)_ to monitor the activity of the *faeB* promoter *via* the ability to grow on acetamide plates or *via* luciferase assay (Reijngoud et al., 2021). Strains were grown on liquid or solidified (containing 1.5% (w/v) Scharlau agar (Scharlau, Barcelona, Spain)) minimal medium (MM) or on complete medium (CM) MM contains 7 mM KCl, 8 mM KH_2_PO_4_, 70 mM NaNO_3_, and 2 mM MgSO_4_ (pH adjusted to 5.5) and spore elements as described (Arentshorst et al., 2012). MM was supplemented with 50 mM carbon source. Complete medium (CM) was also used and consists of MM supplemented with 0.1% casamino acids and 0.5% w/v yeast extract and 50 mM glucose. Transformants of *A. niger* were isolated and purified as described (Arentshorst et al., 2012) using a final concentration of 100 μg/mL hygromycin (*In vivo*Gen, Toulouse France) or 100 μg/mL phleomycin (*In vivo*Gen, Toulouse France). Mycelial growth assays were performed on MM plate, supplemented with 5 mM of the aromatic compounds or with 10 mM fatty acids. Aromatic compounds and fatty acids compounds were weighed, dissolved in sterile water, and mixed with an equal volume of 2 x concentrated MM-agar. Growth of strains was assayed by point inoculation of 5 μL filtered conidium suspension (1 x 10^6^ conidia/mL) in the center of a MM medium containing plate and incubated for 5 to 10 days in the dark at 30°C. Growth characteristics of the strain was analyzed by comparison of the growth of the colony (radial extension, thickness of the mycelium, and the ability to form conidia) over time. Growth assays of different strains on the same substrate were always performed in the same growth experiments for proper comparisons. Growth experiments of the strains were repeated on the different substrate at least twice with consistent results. Growth phenotypes of the mutant strains were compared to the parental strains (MA234.1 or JR11.1) to assess the effects of introduced mutations on carbon source utilization. Growth of the parental strain was scored as ++, and the ++ score for a particular mutants indicates normal growth of the mutant strain compared to the parental strain, + indicates mildly reduced growth of the mutant strain compared to the parental strain, +/– indicates severely reduced growth of the mutant strain compared to the parental strain, and – indicates no growth of the mutant strain (similar as the no-carbon control plate). *Escherichia coli* DH5α was used for plasmid propagation and cultured at 37°C in Lysogeny broth (LB) medium, with ampicillin (100 μg/mL).

**Table 1:** Strains used in this study.

Strain	Genotype	Description	Reference
N402	*cspA1*	Derivative of NRRL3 (N400)	Bos et al., 1988
MA169.4	*cspA1, pyrG378, kusA::DR-amdS-DR*	*ku70* deletion in AB4.1	Carvalho et al., 2010
MA234.1	*cspA1, kusA::DR-amdS-DR*	Restored *pyrG* in MA169.4	Alazi et al., 2016
JR10.2	*PfaeB-amdS, PfaeB-lux*	*PfaeB-lux* in JR9.9	Reijngoud et al., 2021
JR11.1	*PfaeB-amdS, PfaeB-lux*	*PfaeB-lux* in JR9.19	Reijngoud et al., 2021
JR11.1S_7	*PfaeB-amdS, PfaeB-lux*,	UV mutant	This study
JR11.1U_11	*PfaeB-amdS, PfaeB-lux*	UV mutant	This study
JR11.1U_17	*PfaeB-amdS, PfaeB-lux*,	UV mutant	This study
JR11.1U_19	*PfaeB-amdS, PfaeB-lux*	UV mutant	This study
JR11.1U_23	*PfaeB-amdS, PfaeB-lux*	UV mutant	This study
JR11.1U_24	*PfaeB-amdS, PfaeB-lux*	UV mutant	This study
DT6.1	*PfaeB-amdS, PfaeB-lux, farA, phleo*	Ectopically integrated *farA* and *phleo* in JR11.1U#11	This study
DT7.1	*PfaeB-amdS, PfaeB-lux, farD, phleo*	Ectopically integrated *farD* and *phleo* in JR11.1U#17	This study
DT8.1	*cspA1, kusA::DR-amdS-DR, farA::hygB*	*ΔfarA* in MA234.1	This study
DT9.1	*cspA1, kusA::DR-amdS-DR, farB::hygB*	*ΔfarB* in MA234.1	This study
DT10.1	*cspA1, kusA::DR-amdS-DR, farD::hygB*	*ΔfarB* in MA234.1	This study
DT11.1	*cspA1, kusA::DR-amdS-DR, farA::pyrG*	*ΔfarA* in MA169.4	This study
DT12.1	*cspA1, kusA::DR-amdS-DR, farB::pyrG*	*ΔfarB* in MA169.4	This study
DT13.1	*cspA1, kusA::DR-amdS-DR, farD::pyrG*	*ΔfarD* in MA169.4	This study

### Molecular techniques

PCR amplifications were performed using either Phire (diagnostic PCR and bipartite flank amplification) or Phusion High-Fidelity DNA Polymerase (whole gene amplification) (Thermo Fisher Scientific, Carlsbad, CA, USA) according to the instructions provided. All primers are listed in **Supplementary Table 1**. DNA fragments were purified using the GeneJET Gel Extraction Kit (Thermo Fisher Scientific, Carlsbad, CA, USA) and ligations were carried out using the CloneJET PCR Cloning Kit and the Rapid DNA Ligation Kit (Thermo Fisher Scientific, Carlsbad, CA, USA). DNA sequencing of specific genes was performed by Macrogen (The Netherlands).

### Generation of transcription factor mutant library in *A. niger*

The general strategy for constructing and verifying the TF deletion strains is shown in **Supplementary Figure 1**. Primers were designed by Biomax, based on the *A. niger* CBS513.88 genome sequence and available upon request. Deletion constructs with the hygromycin selection marker were generated by PCR and assembled by either Gibson assembly (Gibson assembly cloning Kit, New England Biolabs) or fusion PCR. Gene deletion constructs were transformed into *A. niger* strain MA234.1 (Alazi et al., 2016). When available, two transformants were purified by streaking conidia on MM-hygromycin plates. Gene deletion mutants that gave viable colonies were purified once more on MM-hygromycin plates. For each strain, conidia were isolated from a twice purified clone and used to inoculated cultures to obtain mycelium for genomic DNA isolation and diagnostic PCR. Verification of correct integration was performed using primers as schematically depicted in **Supplementary Figure 1**. Correct integration of the bipartite fragments should lead to the amplification of a PCR fragment as the outside gene specific primer is not present on the bipartite fragment. Initially, 284 transcription factors were selected for gene disruption based on expression data and genomic clustering of transcription factors with potential target genes. In total 239 gene deletion mutants were identified in which at least one transformant was PCR positive for both flanks. Conidia of these transformants were counted and the concentration of conidia was adjusted to 1 x 10^6^ conidia/ml and two glycerol stocks were made for each strain and stored at -80°C. Conidia from the glycerol stock were directly spotted on ferulic acid plates and poor growing strains were retested for growth on ferulic acid. The primers to delete the *farA, farB* and *farD* genes *via* the same method are listed in **Supplementary Table 1**.

### UV mutagenesis

UV mutagenesis experiments were performed as described previously (Damveld et al., 2008) using JR11.1 (*PfaeB-amdS, PfaeB-lux_613_*) as the starting strain (Reijngoud et al., 2021). After determining the killing rate of the UV treatment, conidia with 60-70% survival were plated out on fluoro-acetamide plates with 0.05% ferulic acid. On each plate about 5*10^5^ conidia (50 µL of 1*10^7^ conidia/mL suspension) were plated. In total, around 30 plates for JR11.1 were prepared and incubated for 7 days at 30°C. Two plates were included on which non-mutagenized conidia were plated out. Mutants growing and sporulating on the fluoro-acetamide plates were purified twice. In total, six mutants resistant to fluoro-acetamide were isolated and analyzed in more detail for growth on different carbon sources (sugars, aromatics and fatty acids) and in luciferase assays.

### Luciferase assays

Luciferase (lux) assays were performed as follows: 176 μL of MM (containing 1% (w/v) fructose and 0.003% (w/v) yeast extract), 4 μL 25 mM luciferin (Promega, E1605, Leiden, The Nethrlands) and 20 μL conidium suspension (1*10^6^ conidia/mL) were pipetted together (in triplicate) into a well of a white, clear bottom, 96- well plate (Greiner Bio-One, ref. 655095, Alphen aan den Rijn, The Netherlands) and incubated for 24 h at 30°C in the Spark 10M Multimode Microplate reader (Tecan) while measuring luciferase activity and OD_600_ every 15 min. MM containing 0.1% (w/v) ferulic acid was prepared by dissolving 0.25 g ferulic acid in 250 mL MM, followed by filter sterilization. MM with lower concentrations of ferulic acid was prepared by diluting MM containing 0.1% ferulic acid with appropriate volumes of MM.

### Sequencing of *farA*

To sequence the *farA* gene in JR11.1 derived mutants, the *farA* gene was amplified using Phire polymerase (high fidelity DNA polymerase, Thermo Fisher Scientific, Carlsbad, CA, USA) from genomic DNA of the respective mutant using primers FarAP1 and FarAP18 (**Supplementary Table 1**). The amplified 5.6- kb *farA* gene including ~1-kb flanking regions was sequenced using primers listed in **Supplementary Table 1**. Sequence reads were aligned to the *farA* gene of NRRL3 retrieved from gb.fungalgenomics.ca/portal/and analyzed in DNAman.

### Genome sequencing of JR11.1 and JR11.1_U17, JR11.1_U19, and JR11.1_U24

Genomic DNA of *A. niger* strains was isolated as described (Arentshorst et al., 2012). The genomic DNA was further column-purified (NucleoSpin Plant II, Macherey-Nagel) for whole genome sequencing. The genomes of were sequenced at the McGill University Quebec Innovation Centre (Montreal, Canada) using the Illumina HiSeqX platform to about 125-fold coverage. The raw DNA reads, as received from the sequencing centre, were mapped to the *Aspergillus niger* NRRL3 v1 assembly with bowtie2 (Langmead and Salzberg, 2012). Variants were called with pilon (Walker et al., 2014) version 1.22 in –variants mode. The VCF files produced by pilon were filtered with vcffilter (Garrison et al., 2021) to keep only variants at locations covered by 6 or more reads, with no more than two genotypes present, and with more than 80% of the covering reads bearing the variant genotype. Variants at the same locations in the filtered files from all samples were tabulated with a custom Python script. Variants inside the coding sequence of a predicted gene and their effect on the predicted protein sequence were identified with another Python script. The Sequence Read Archive accession numbers for the JR11.1 parental strain and derived mutants JR11.1_U17, JR11.1_U19, and JR11.1_U24 are SRR18932555, SRR18932554, SRR18932553, and SRR18932552, respectively.

### Complementation analysis of *farA* and *farD* mutants

Primers for PCR were designed to amplify complete expression cassettes for *farA* and *farD* open reading frames, including a ~1000 base pair promoter region and a ~500 base pair terminator region. PCR fragments were ligated into pJet1.2 (Thermo Fisher Scientific, Carlsbad, CA, USA) and sequenced. pJet1.2 plasmids containing the *farA* and *farD* genes were co-transformed to JR11.1_U11 (*farA*) and JR11.1_U17 (*farD*) with pAN8.1 (Punt and van den Hondel, 1992) which allows selection on phleomycin. Transformants were purified on solidified MM-phleomycin plates (100 μg/mL phleomycin) and single colonies were propagated on CM to harvest conidia. Conidia were spotted on MM-ferulic acid plates as described above. FarA and FarD primers (**Supplementary Table 1**) were used to amplify respective genes and PCR fragments were directly sequenced to confirm the presence of both the wild type and mutant allele in the respective complemented strains.

### Phylogenetic analysis

Protein sequences from the *A. niger* NRRL3 strain encoding FarA (NRRL3_00665), FarB NRRL3_06630 and FarD (NRRL3_1945) were initially retrieved from https://gb.fungalgenomics.ca/portal/. The protein sequence from the *A. flavus* FarC protein (Aspfl2_3|2227896) was retrieved from https://mycocosm.jgi.doe.gov/mycocosm/home. Proteins sequences were used as queries for BLASTP searches at https://blast.ncbi.nlm.nih.gov/Blast.cgi?PAGE=Proteins. Hits were manually analyzed and ordered in their respective Far-family. For the 12 fungal species included in the phylogenetic tree, protein sequences were retrieved from https://mycocosm.jgi.doe.gov/mycocosm/home. Retrieved protein sequences were aligned in Clustal omega (https://www.ebi.ac.uk/Tools/msa/clustalo/). Upon the suspicion of misalignment due to structural annotation issues (false prediction of start/stop codon or intron/exon bounderies), genomic sequences were retrieved from the JGI website (https://mycocosm.jgi.doe.gov/mycocosm/home) and manually inspected and altered to improve the genemodel and thereby improving aligment of the protein sequences. The final phylogenic tree included in this study is based on those improved gene models.

## Results

### The FarA and FarB Zn(II)_2_Cys_6_transcription factors are involved in ferulic acid metabolism in *A. niger*

To identify transcription factors (TFs) required for growth on ferulic acid, a collection of 239 TF mutants was tested for growth on 0.2% (10.3 mM) ferulic acid. The genes encoding the TFs that were deleted are listed in **Supplementary Table S2**. The screen yielded two TF mutants with a reduced growth phenotype on ferulic acid, namely a deletion mutant in which the gene encoding the AcuK transcription factor was deleted *(ΔacuK*), and a deletion mutant in which the gene encoding the FarA transcription factor was deleted *(ΔfarA*). AcuK is required for the expression of genes related to gluconeogenesis (Suzuki et al., 2012). Because aromatics like ferulic acid are likely to be metabolized *via* TCA cycle intermediates, gluconeogenesis is key in sustaining growth using these compounds. The strongly reduced growth of the *ΔacuK* mutant on ferulic acid is therefore most likely not directly related to ferulic acid metabolism itself and the *ΔacuK* mutant was not studied further. The FarA transcription factor has an established function in fatty acid metabolism in *A. nidulans* (Hynes et al., 2006) and since ferulic acid metabolism was recently found to be dependent on the peroxisomal CoA-dependent β-oxidative genes (Lubbers et al., 2021a) the possible link of *farA to* ferulic acid metabolism was further investigated.

**Figure 2:**
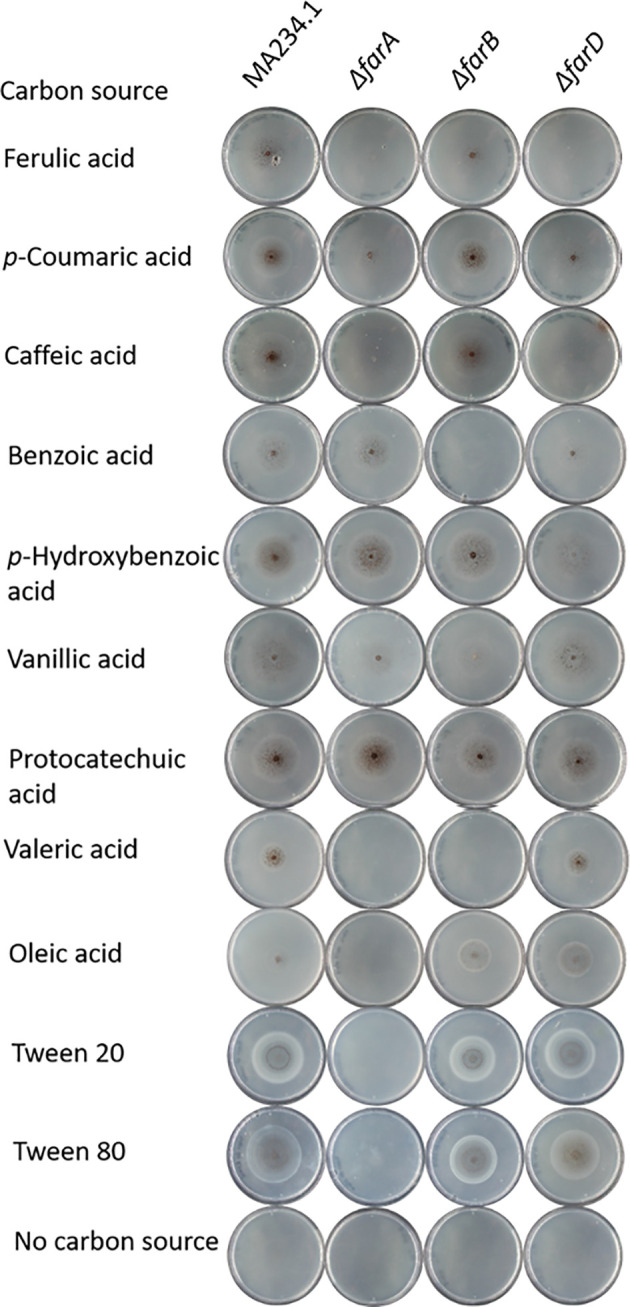
Phenotypic analysis of *ΔfarA, ΔfarB* and *ΔfarD* mutants. Mutant were grown for seven days on minimal medium with 5mM hydroxycinnamic acids, 5 mM benzoic acid derivatives, 1 mM valeric acid and 5 mM long chain fatty acids. Strain phenotypes were compared to the MA234.1 strains to assess the effects of introduced mutations on carbon source utilization.

A second fatty acid regulator present in aspergilli is the FarB transcription factor. In *A. nidulans*, FarB has been shown to be required for the metabolism of short chain fatty acids (Hynes et al., 2006). Since a correct *farB* deletion mutant was not present in the original collection, a Δ*farB* deletion mutant was made. The correct deletion of *farA* and *farB* in the respective mutants was confirmed by diagnostic PCRs (**Supplementary Figures 2** and **3**). The growth analysis on ferulic acid showed that deletion of *farA* strongly reduced growth on ferulic acid, while deletion of *farB* showed a partially reduced growth and reduced sporulation phenotype on ferulic acid (**Figure 2**). A more detailed growth analysis of the *ΔfarA* and *ΔfarB* mutants in addition to other mutants and other substrates will be presented below. A third Far-like transcription factor (FarC) has been identified in a number of Aspergilli, including *Aspergillus flavus* (Luo et al., 2016) but a FarC homolog is not present in the genome of *A. niger* (see below).

### Isolation of *trans*-acting mutants unable to induce *faeB* expression under inducing conditions

**Figure 3:**
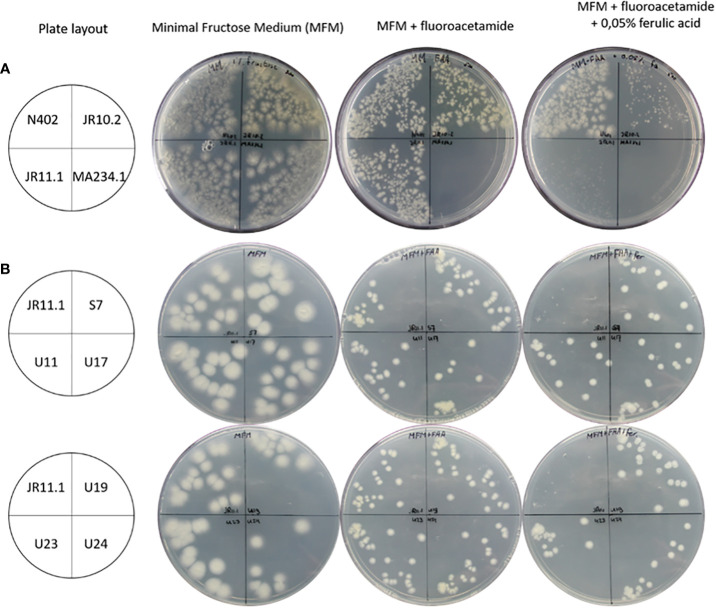
Growth analysis of reporter strains and reporter strain derived mutants. **(A)** Analysis of strains JR10.2 and JR11.1 for susceptibility to 5-fluoro-acetamide in the presence of the ferulic acid inducer. Growth of the reporter strains was compared to N402 and MA234.1 (*amdS* gene expressed from the constitutive *gpdA* promoter) on minimal fructose medium (MFM), MFM supplemented with 0.2% 5-fluoro-acetamide and MFM supplemented with 0.2% 5-fluoro-acetamide and 0.05% (2.6 mM) ferulic acid. Roughly 500 conidia of each strain were spread on the plates in the indicated quadrants. **(B)** Screen for UV mutants in the JR11.1 background that are resistant to fluoro-acetamide in the presence of the ferulic acid inducer. Growth of six UV mutants was compared to the JR11.1 parental strain on minimal fructose medium (MFM), MFM supplemented with 0.2% fluoro-acetamide and MFM supplemented with 0.2% fluoro-acetamide and 0.05% (2.6 mM) ferulic acid. Roughly 20 conidia of each strain were spread on the plates in the indicated quadrants.

To identify additional regulatory mutants unable to induce the ferulic acid esterase B gene (*faeB*) in response to the presence of ferulic acid, a positive forward genetic screen was set up. The screen is based on the use of a *PfaeB-amdS*, *PfaeB-lux_613_* dual reporter strain (Reijngoud et al., 2021) to isolate mutants unable to induce the *faeB* gene. The rationale of the screen is that ferulic acid will induce expression of the *PfaeB-amdS* reporter and that expression of the *amdS* gene will result in lethality on plates containing fluoro-acetamide. Mutants unable to induce the *amdS* gene from the *PfaeB* promoter will be resistant to fluoro-acetamide. The principle of the screen was assessed by analyzing the growth of *PfaeB-amdS*-containing reporter strains JR10.2 and JR11.1 (Reijngoud et al., 2021) on fluoro-acetamide plates in the absence and presence of ferulic acid. Induction of the *PfaeB-amdS* reporter in strain JR11.1 and to a lesser extent in JR10.2 by ferulic acid resulted in reduced growth on fluoro-acetamide plates (**Figure 3**). The growth reduction of reporter strain JR10.2 on fluoro-acetamide was less severe than of JR11.1 and is probably due to the presence of multiple copies of the *pfaeB-amdS* reporter in JR11.1. Because of the more reduced growth of JR11.1 on fluoro-acetamide plates containing ferulic acid, this strain was used for the isolation of mutants. To obtain mutants JR11.1 conidia were UV-mutagenized and plated out on MM medium plates containing fructose and nitrate as carbon and nitrogen sources respectively and 0.2% fluoro-acetamide and 0.05% ferulic acid. In addition, conidia that were not mutagenized were also plated out to isolate spontaneous mutants. From the initial screening plates 29 colonies were purified twice on MM plates containing fluoro-acetamide and ferulic acid, which resulted in the isolation of six mutants that were resistant to fluoro-acetamide in the presence of ferulic acid. One spontaneous mutant of JR11.1 was obtained (JR11.1_S7) and five mutants were obtained after UV mutagenesis (JR11.1_U11, JR11.1_U17, JR11.1_U19, JR11.1_U23 and JR11.1_U24. The ability to grow on fluoro-acetamide plates in the presence of 0.05% ferulic acid of these six JR11.1-derived UV mutants is shown in **Figure 3**.

**Figure 4:**
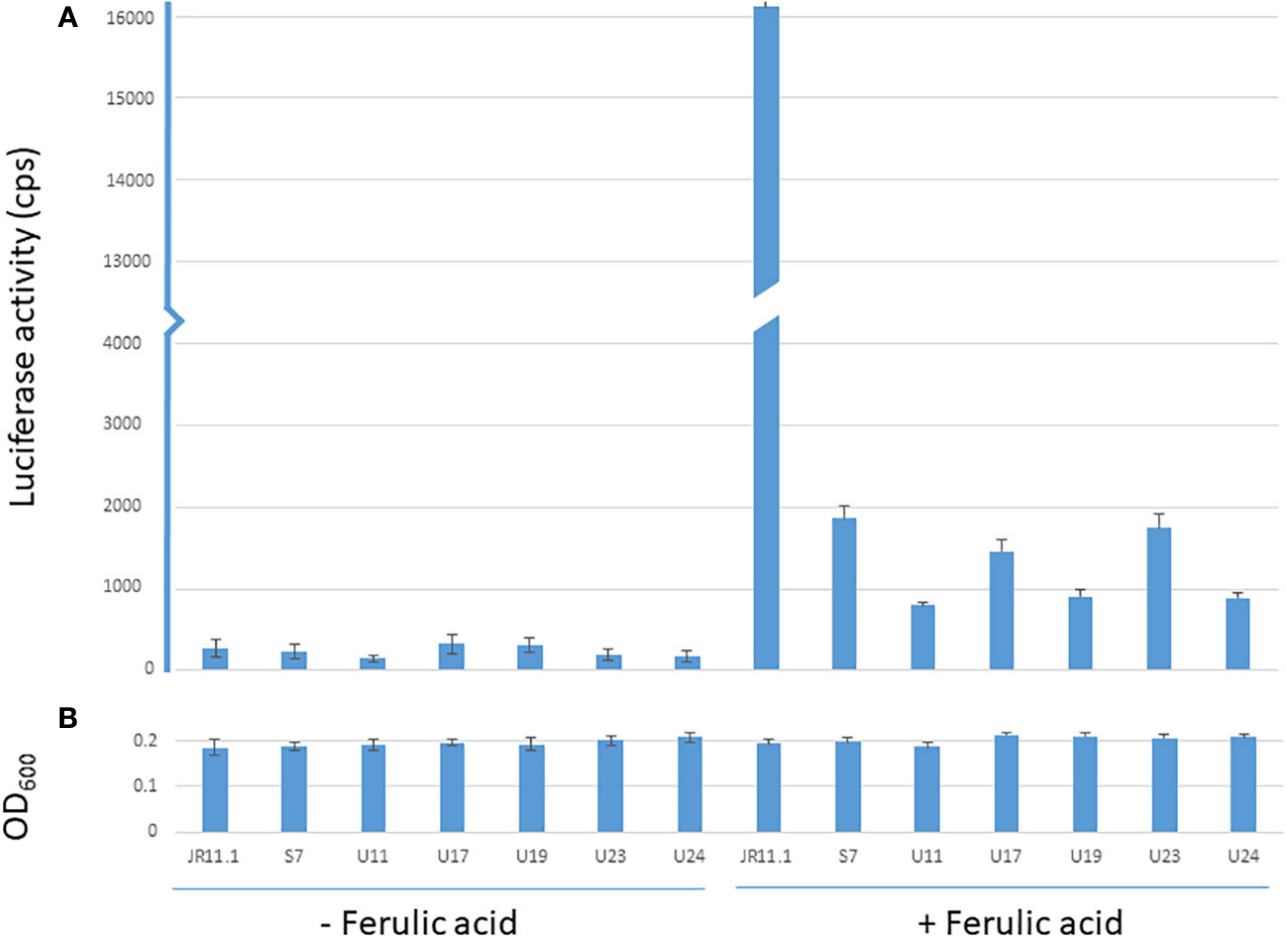
Luciferase and OD_600_ values of the reporter strain and mutants under non-inducing and inducing conditions. Luminescence values (cps) of reporter strain JR11.1 and mutants derived from JR11.1 (S7, U11, U17, U19, U23 and U24) were compared to parental strain JR11.1. Strains were grown in MM with D-fructose as carbon source without or with 5 mM ferulic acid as inducer. Luciferase activity **(A)** and OD_600_ values **(B)** after 20 hours of growth at 30 °C are shown.

The JR11.1 strain also carries the *PfaeB-luciferase* (*PfaeB-lux_613_*) reporter construct. To determine if the mutations that allow these six mutants in the JR11.1 background to grow on fluoro-acetamide were *trans*-acting, luciferase assays under non-inducing and inducing conditions were performed. Expression of the *pfaeB-lux_613_* reporter is low under non-inducing conditions and was not induced by 5 mM ferulic acid in the six mutants while ferulic acid strongly induces luciferase activity in the JR11.1 reporter strain (**Figure 4**). This indicates that the mutations introduced in the fluoro-acetamide resistant mutants work in *trans*; the mutations affect both the *faeB* promoter upstream of the *amdS* gene and the *faeB* promoter upstream of the *lux_613_* gene.

### Growth analysis of *ΔfarA*, *ΔfarB* and the six *trans*-acting fluoro-acetamide resistant mutants for growth on aromatics and fatty acid carbon sources

With the confirmation that the six fluoro-acetamide resistant mutants contained mutations functioning in *trans*, a detailed growth analysis on a variety of carbon sources including hydroxycinnamic acids, benzoic acid-derivatives, and long- and short fatty acids was performed with the *ΔfarA* and *ΔfarB* mutants (**Figure 1** and **Table 2**) and the six mutants (**Table 2**). All six mutants displayed a reduced growth phenotype on ferulic acid, caffeic acid and coumaric acid, similar to the *ΔfarA* strain. The *ΔfarB* strain showed a reduced growth phenotype on ferulic acid compared to the parental strain, but the growth defect was less severe than that found for the *ΔfarA* strain.

**Table 2:** Growth characteristics of *ΔfarA*, *ΔfarB*, *ΔfarD* and JR11.1-derived mutants.

Strain name	Hydroxycinnamic acids			benzoic acid derivatives				Short chain fatty acids	Long chain fatty acids		
	Fer	Cou	Caf	Van	pHB	PCA	Ben*	Val	Ole*	TW20	TW80
MA234.1	++	++	++	++	++	++	++	++	++	++	++
*ΔfarA*	+/-	+/-	+/-	++	++	++	++	-	+/-	-	-
*ΔfarB*	+	++	++	+	++	++	+	-	++	++	++
JR11.1	++	++	++	++	++	++	++	++	++	++	++
JR11.1_S7	+/-	+/-	+/-	++	++	++	++	-	+/-	-	-
JR11.1_U11	+/-	+/-	+/-	++	++	++	++	-	+/-	-	-
JR11.1_U17	+/-	+/-	+/-	++	+/-	+	++	++	++	++	++
JR11.1_U19	+/-	+/-	+/-	++	+/-	+	++	++	++	++	++
JR11.1_U23	+/-	+/-	+/-	++	++	++	++	-	+/-	-	-
JR11.1_U24	+/-	+/-	+/-	++	+/-	+	++	++	++	++	++
											
*ΔfarD*	+/-	+/-	+/-	++	+/-	+	++	++	++	++	++

Phenotypic analysis of parental strains (MA234.1 and JR11.1), *ΔfarA*, *ΔfarB* mutants and JR11.1-derived mutants after seven days of growth on minimal medium supplemented with 5 mM carbon sources. Growth on the following groups of carbon sources was examined: hydroxycinnamic acids (Fer – ferulic acid; Cou – p-coumaric acid; Caf – caffeic acid), benzoic acid derivatives (pHB – p-hydrobenzoic acid; Ben – benzoic acid; Van – vanillic acid; PCA – protocatechuic acid), short chain fatty acids (Val – valeric acid) and long chain fatty acids (Ole – oleic acid; TW20 – Tween 20; Tw80 – Tween 80). Growth phenotypes of the mutant strain were compared to the parental strains (MA234.1 or JR11.1) to assess the effects of introduced mutations on carbon source utilization. Growth of the parental strain was scored as ++, and the ++ score for a particular mutants indicates normal growth of the mutant strain compared to the parental strain, + indicates mildly reduced growth of the mutant strain compared to the parental strain, +/– indicates severely reduced growth of the mutant strain compared to the parental strain, and – indicates no growth of the mutant strain (similar as the no-carbon control plate). Reduced growth of the strains is highlighted in gray. *Note that the growth of *A. niger* parental strains on benzoic acid and oleic acid is already poor and further reduced in some mutants as indicated.

The *ΔfarA* and *ΔfarB* strains and the six fluoro-acetate resistant mutants were tested for growth on the benzoic acid relatives of hydroxycinnamic acids. The benzoic acid derivatives have the same ring structure as hydroxycinnamic acids, but lack their C3-moiety (prop-2-enoic acid (-C=C-COOH)). Growth on these carbon sources revealed two groups within the six mutants. The first group consists of the *ΔfarA* mutant and mutants JR11.1_S7, JR11.1_U11 and JR11.1_U23, which were able to grow on all tested benzoic acid derivatives (p-hydrobenzoic acid and benzoic acid, vanillic acid and protocatechuic acid) whereas the second group, consisting of JR11.1_U17, JR11.1_U19 and JR11.1_U24, showed a reduced growth phenotype on *p*-hydroxybenzoic acid. The *ΔfarB* strain showed a growth reduction on benzoic acid (**Figure 1** and **Table 2**).

The growth analysis of the different mutants on short- and long chain fatty acids revealed that mutants JR11.1_S7, JR11.1_U11 and JR11.1_U23 showed a reduced growth phenotype on short fatty acids (valeric acid) and long chain fatty acids (oleic acid, Tween 20 and Tween 80) similar to the *ΔfarA* mutant. Mutants JR11.1_U17, JR11.1_U19 and JR11.1_U24 were able to grow on short- and long chain fatty acids, indicating that these mutants phenotypically differ from *ΔfarA* mutants. The *ΔfarB* mutant did not grow on the short fatty acid tested, but grew well on long fatty acids (**Figure 1** and **Table 2**), indicating that mutants JR11.1_U17, JR11.1_U19 and JR11.1_U24 were also different from *farB* mutants.

### Genetic characterization of ferulic acid non-utilizing mutants

**Figure 5:**
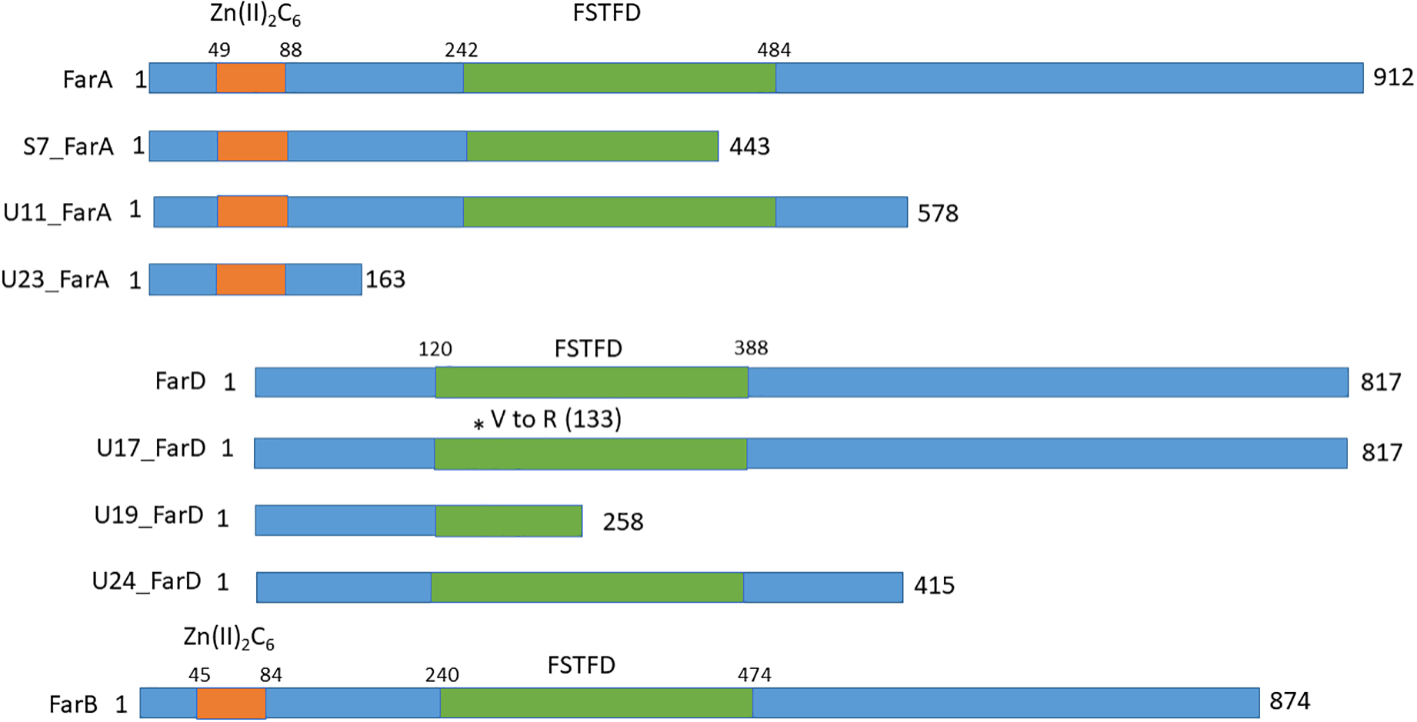
Schematic overview of the domains and the positions of nonsense mutations in FarA, FarD and FarB identified in the fluoro-acetamide resistant mutants. The Zn(II)2Cys6 binding domain in FarA and FarB are indicated by the orange box and the Fungal Specific Transcription Factor Domain (FSTFD) is indicated in green. * indicates the position of the mutation.

Since the growth phenotypes of three of the six fluoro-acetamide mutants was identical to the growth phenotype of the *ΔfarA* mutant, the *farA* gene was amplified and sequenced for these three mutants (JR11.1_S7, JR11.1_U11 and JR11.1_U23) as well as for the remaining three mutants (JR11.1_U17, JR11.1_U19 and JR11.1_U24). Sequencing analysis revealed that the three *farA*-like mutants (JR11.1_S7, JR11.1_U11 and JR11.1_U23) contain mutations in the *far*A gene, while no mutations were found in the *farA* gene of the three other mutants (JR11.1_U17, JR11.1_U19 and JR11.1_U24). Mutant JR11.1_S7 has a T to G mutation, which leads to nonsense mutation TAT (Tyrosine) to TAG (stop codon) at nucleotide position 2063 in the *farA* ORF. The resulting truncated FarA protein has only 443 amino acids of the 912 amino acids remaining. JR11.1_U11 has a T to A mutation leading to a nonsense mutation TTA (Leucine) to TAA (stop codon) at nucleotide position 3102 in the *farA* ORF, resulting in a truncated protein of 578 amino acids. In mutant JR11.1_U23 a T has been inserted at nucleotide position 1092, resulting in a frameshift and an immediate nonsense mutation CCT AAG (Proline and Lysine) to CCT TAA (Proline and Stop codon) at amino acid position 163. The presence of a nonsense mutation in the *farA* gene of all three of the mutants and the fact that all three mutants have the same phenotype suggests a loss-of-function mutation of *farA*, which corresponds with the observed *ΔfarA*-like phenotype. FarA contains two PFAM domains including a Zn(II)_2_Cys_6_ binuclear cluster domain (PF00172.18) comprising amino acids 49-88 and a fungal specific transcription factor domain (PF04082.18) comprising amino acids 242-484. The position of these domains as well as the positions of nonsense mutations in the mutants is shown in **Figure 5**. The loss-of-function mutation in JR11.1-U11, leading to a C-terminally truncated version of FarA, indicates that the Zn(II)_2_Cys_6_ binuclear cluster domain and the fungal specific transcription factor are not sufficient for a functional FarA protein.

To identify the gene(s)s responsible for the reduction of growth on ferulic acid shown for the three remaining mutants (JR11.1_U17, JR11.1_U19 and JR11.1_U24), whole genome sequencing was performed. All three mutants showed only a limited number of SNPs in open reading frames, with JR11.1_U17, JR11.1_U19 and JR11.1_U24 having 6, 9, and 6 SNPs respectively in protein-encoding regions (**Supplementary Table 3**). All three mutants contain a mutation in the gene *NRRL3_09145*, which is predicted to encode a protein of 817 amino acids. A PFAM analysis showed that the protein encoded by *NRRL3_09145* contains a fungal specific transcription factor domain (PF04082.18) comprising amino acids 120-388. Usually this domain is accompanied by a Zn(II)_2_Cys_6_ binuclear cluster domain. However, in the case of NRRL3_09145, this domain was absent. Incorrect annotation of the predicted region was carefully checked by computationally and manually analyzing the DNA sequence and mRNA transcripts of *NRRL3_09145*. The mRNA reads corresponding to the *NRRL3_09145* from several growth conditions showed no evidence of alternative splicing; e.g. an unusually long intron, that could explain the absence of a Zn(II)_2_Cys_6_ binuclear cluster domain in the so far annotated gene model (data not shown). The Zn(II)_2_Cys_6_ binuclear cluster domain was absent in *A. niger* and other filamentous fungi that possess a NRRL3_09145 orthologue (see below). Using NRRL3_09145 as a query for a BLASTP search to look for homologous proteins in *A. niger* revealed that NRRL3_09145 is most similar to the FarB transcription factor and more distantly related to FarA. The FarB protein shows 24% identity over the entire protein sequence to FarD, while FarA and FarD show 16% identity towards each other. The identity between FarB and FarD is mostly found in the fungal specific transcription factor domain (29%) and FarA and FarD show 17% identity in the fungal specific transcription factor domain. Far A and FarB show 16 and 19% sequence identity between the entire protein sequence and the fungal specific transcription factor domain, respectively. Since the name FarC was already given to a FarA/B like protein found in *A. flavus* and some other Aspergilli, and because NRRL3_09145 is clearly different from the FarC proteins, the protein encoded by the *NRRL3_09145* gene is called FarD.

At least two of the mutations found in *farD* suggest that the observed phenotype of the mutants is due to a loss of function of *farD* (**Figure 5**). JR11.1_U17 has a C to T mutation at position 1402 in the *farD* ORF, leading to a nonsense mutation CAA (Q) to TAG (Stop codon) and resulting in a truncated protein of 451 amino acids. JR11.1_U19 has GT to AG mutations leading to a missense mutation GTG (Valine) to AGG (Arginine) at nucleotide position 396/397 in the *farD* ORF, resulting in an amino acid change at position 133. JR11.1_U24 has an A to T mutation at position 826 in the *farD* ORF, leading to a nonsense mutation AAG (Leucine) to TAA (Stop codon) resulting in a truncated protein of 258 amino acids.

To confirm that the observed phenotypes of the two identified groups of UV mutants were indeed caused by loss-of-function mutations in *farA* or *farD*, mutants JR11.1_U11 (*farA*) and JR11.1_U17 (*farD*) were complemented by transformation with a full length version of *farA* or *farD* gene expressed from their endogenous promoter. Complemented strains were able to grow on ferulic acid indicating that a loss-of-function of *farA* or *farD* is responsible for the ferulic acid related phenotypes observed in JR11.1_U11 and JR11.1_U17, respectively.

### Targeted deletion of *farD* confirms its role in ferulic acid metabolism

To further confirm the role of *farD* for growth on ferulic acid as well as on other substrates, the *farD* gene was deleted in MA234.1 using the hygromycin split marker. Proper deletion of the *farD* gene was confirmed by diagnostic PCR (**Supplementary Figure 4**). The phenotypic growth defect of the Δ*farD* mutant was compared to the growth phenotype of the Δ*farA* and Δ*farB* mutants as well as the *farD* mutants resulting from the UV mutagenesis. As expected, deletion of *farD* led to the growth phenotype first observed for the *farD* UV mutants (**Figure 1** and **Table 2**).

Growth phenotypes of the *ΔfarA*, *ΔfarB* and *ΔfarD* strains on various substrates are shown in **Figure 1** and summarized in **Table 2**. From these results it is clear that both FarA and FarD are important for growth on ferulic acid, coumaric acid and caffeic acid. Importantly, while the function of FarA and FarD is required for growth on these hydroxycinnamic acids, FarA and FarD seem to have distinct functions in the metabolism of short and long chain fatty acids. As shown, FarA is required for fatty acid metabolism, while FarD is fully dispensable for growth on valeric acid, oleic acid, Tween20 and Tween80 (**Figure 2**). FarB is specifically required for growth on the short chain fatty acid valeric acid, but not for growth on long chain fatty acids (**Figure 2**).

### Phylogenetic analysis of the family of Far transcription factors

Several Far transcription factors have been previously described and studied. FarA and FarB are relatively well studied in a few species (mainly *A. nidulans*, *A. oryzae*, *A. flavus* and *Fusarium oxysporum*) (Hynes et al., 2006, Rocha et al., 2008; Garrido et al., 2012; Luo et al., 2016; Bermúdez-García et al., 2019) In *A. flavus*, another homolog of FarA and FarB was identified and named FarC (Luo et al., 2016). The gene model of *A. flavus* FarC was updated compared to the initial publication and predicted to consist of 527 amino acids (**Supplemental Data 1**). In this study, an additional member of the Far transcription factor family was identified and named FarD.

The *farD* gene of *A. niger* is predicted to encode a 817 amino acid long protein. Protein BLAST searches using the *A. niger* FarD protein (NRRL3_09145) as a query revealed that the protein is conserved among fungal species within the order Eurotiales. Fungi belonging to the same class of Eurotiomycetes, but a different order (Onygenales) which include *Coccidioides immitis*, *Trichophyton rubrum*, and *Histoplasma capsulatum*, do not contain a FarD ortholog. More distantly related Pezizomycetes fungi belonging to the classes of Leotiomycetes, Sordariomycetes, and Dothideomycetes also do not have a FarD homolog. In **Table 3**, we have summarized the presence or absence of FarD homologs in a selection of 28 fungal species representing the Pezizomycotina group. None of the FarD orthologs identified contained a Zn(II)_2_Cys_6_ DNA binding domain, but all contained the fungal specific transcription factor domain (PFAM PF04082). The gene model of *A. oryzae*, the only aberrant model, is predicted to be 163 amino acids longer at the N-terminus of the protein. However, this predicted protein sequence also does not contain a Zn(II)_2_Cys_6_ DNA binding domain and is likely a result of a mispredicted gene model.

**Table 3:** Presence of FarA, FarB and Far-like protens TF in 28 Pezizomycotina species.

Family	Strain	FarA	FarB	FarC	FarD
Eurotiomycetes	*Aspergillus niger* NRRL3	1	1	o	1
Eurotiales	*Aspergillus carbonarius* ITEM 5010	1	1	o	1
	*Aspergillus flavus* NRRL3357	1	1	1	1
	*Aspergillus terreus* NIH2624	1	1	1	1
	*Aspergillus fumigatus* Af293	1	1	1	1
	*Aspergillus oryzae* RIB40	1	1	0	1
	*Aspergillus glaucus* CBS 516.65	1	1	1	1
	*Aspergillus clavatus* NRRL 1	1	1	1	1
	*Aspergillus nidulans* FGSC A4	1	1	0	1
	*Aspergillus zonata* CBS 506.65	1	1	0	1
	*Talaromyces stipitatus* ATCC 10500	1	1	0	1
	*Penicillium rubens* Wisconsin 54-1255	1	1	0	1
Eurotiomycetes	*Coccidioides immitis* RS	1	1	0	0
Onygenales	*Trichophyton rubrum CBS 118892*	1	1	0	0
	*Histoplasma capsulatum* NAm1	1	2	0	0
Leotiomycetes	*Botrytis cinerea* T4	1	1	0	0
	*Sclerotium cepivorum*	1	1	0	0
	*Sclerotinia sclerotiorum* 1980 UF-70	3	1	0	0
	*Marssonina brunnea* _m1	1	1	0	0
Sardariomycetes	*Trichoderma reesei* QM6a	1	1	0	0
	*Neurospora crassa* OR74A	1	1	0	0
	*Thermothelomyces thermophilus* ATCC 42464	1	1	0	0
	*Pyricularia oryzae* Y34	1	1	0	0
	*Fusarium graminearum* PH-1	1	1	0	0
	*Fusarium oxysporum* NRRL 32931	1	1	0	0
Dothideomycetes	*Zymoseptoria* tritici IPO323	1	1	0	0
	*Bipolaris maydis* ATCC 48331	1	1	0	0
	*Aureobasidium pullulans* EXF-150	1	1	0	0

FarA and FarB orthologs are found in all 28 species analyzed (**Table 3**). Using the newly predicted gene model of *A. flavus* FarC as a query, only a few species, all belonging to the order Eurotiales, contained a ortholog of FarC (**Table 3**). *Aspergillus flavus* FarC as well as any of the other FarC homologs do not contain a Zn(II)_2_Cys_6_ domain but all contain the fungal specific transcription factor domain (PFAM PF04082).

**Figure 6:**
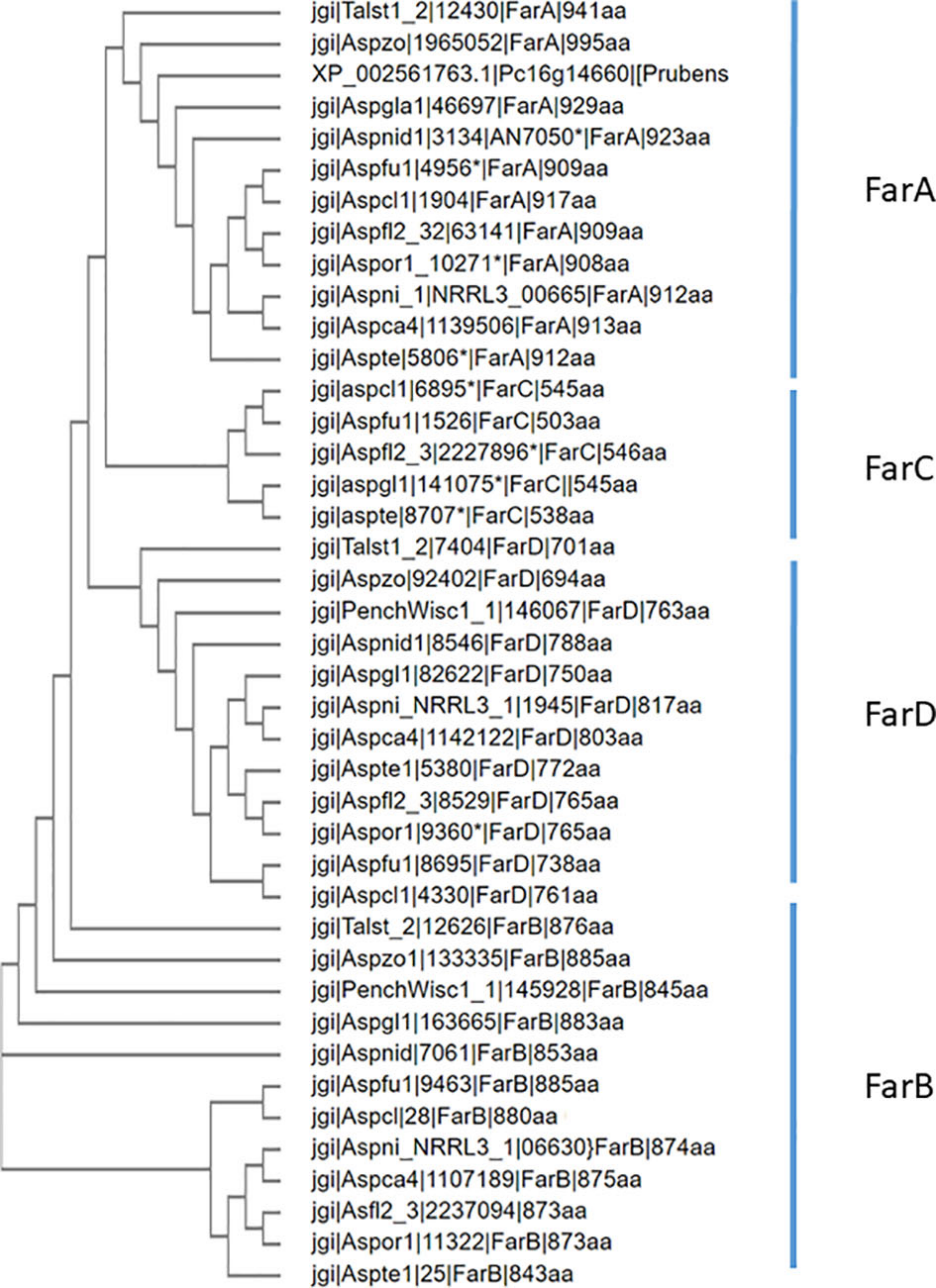
Phylogenetic analysis of Far transcription factors and Far-like transcription factor in the genomes of 12 representative Eurotiales fungal genomes. Using Far proteins from *A. niger* (FarA, FarB, FarD, NRRL3_11483 and NRRL3_07314 or *A. flavus* (FarC) as queries, orthologous Far-like transcription factors were identified. Clustal omega was used to construct the phylogenetic tree.

For the 12 species belonging to the Eurotiales that are mentioned in **Table 3**, a phylogenetic tree was constructed using Clustal Omega for the FarA, FarB, FarC and FarD proteins (when present). To optimize protein alignments, gene models were optimized to include e.g. a Zn(II)_2_Cys_6_ domain or to adjust intron/exon bouderies if appropriate. The protein sequences used to construct the phylogenetic tree are listed in **Supplementary Data 1**. The phylogenetic analysis shows that the Far protein form distinct phylogenetic (**Figure 6**).

## Discussion

The interest in fungal metabolic pathways required for the degradation of plant derived aromatic compounds has increased a lot in the last decade, but detailed knowledge is still limited (see for recent reviews Mäkelä et al., 2015; Lubbers et al., 2019a). One of the reasons for the interest is related to the transition from a fossil-based economy to a bio-based economy using plant cell wall carbohydrates for the production of biofuels and chemicals. Plant cell walls are mostly composed of polysaccharides (cellulose, xylan, arabinanan and pectins) but also contain significant amounts of lignin (Liu et al., 2018). The amount of lignin in the plant cell wall varies among species and the plant tissue and varies between 1 and 24% of lignin of total cell wall residues (Neutelings, 2011) Lignin is generally a polymer composed of three main types of monolignol building blocks (sinapyl alcohol, coniferyl alcohol, and p-coumaryl alcohol) present in plant secondary cell walls (see for review Liu et al., 2018). Although *A. niger* does not possess the enzyme spectrum to fully degrade lignin, it can grow on and metabolize the aromatics of which lignin consists. Apart from lignin, plant cell walls contain other aromatic compounds such as tannins and hydroxycinnamic acids like ferulic acid which *A. niger* can metabolize (van Diepeningen et al., 2004; Lubbers et al., 2019b; Lubbers et al., 2021a; Arentshorst et al., 2021). Another reason for the interest in improving knowledge about enzymes involved in the metabolism of aromatic compounds is to make valuable compounds from the left-over lignins or other plant cell associated aromatics (Kamimura et al., 2019; Lubbers et al., 2021b) obtained from biorefinery processes. In the last few years several metabolic pathways for aromatics have been (partially) elucidated in Aspergilli, including salicylic acid (Martins et al., 2015; Lubbers et al., 2021c), protocatechuic acid (Martins et al., 2015; Lubbers and de Vries, 2021), ferulic acid (Lubbers et al., 2021a) and gallic acid (Arentshorst et al., 2021). Ferulic acid represents an important aromatic compound in relation to efficient conversion of plant cell wall biomass into fermentable sugars. The pathway of ferulate metabolism has been recently studied and several genes involved in the peroxisomal CoA-dependent β-oxidative pathway were found to be required for the degradation of hydoxycinnamic acids, including ferulic acid, p-coumaric acid and caffeic acid (Lubbers et al., 2021a). The peroxisomal CoA-dependent β-oxidative pathway is wellknown to be required for the metabolism of fatty acids in fungi (Hiltunen et al., 2003; Hynes et al., 2008) and is also essential for hydroxycinnamic acid catabolism.

The transcriptional control of genes involved in metabolic degradation of aromatics is largely unknown. Only a few transcription factors that are involved in regulation of genes encoding the enzymes involved the degradation of aromatics have been identified. These include the Zn(II)_2_Cys_6_ transcription factor SdrA which is required for the metabolism of cinnamic acid and sorbic acid (Plumridge et al., 2010; Lubbers et al., 2019b, Seekles et al., 2022) and the TanR Zn(II)_2_Cys_6_ transcription factor required for the degradation of tannin into gallic acid and subsequent metabolism of gallic acid (Arentshorst et al., 2021). Here we show that deletion of FarA, a well described transcription factor required for the induction of genes involved in peroxisomal degradation of fatty acids (Hynes et al., 2006; Hynes et al., 2008), is also required for the metabolism of hydroxycinnamic acids, including ferulic acid, p-coumaric acid and caffeic acid. The degradation of cinnamic acid was less affected in our mutants (results not shown), because its metabolism uses a different pathway controlled by the SdrA transcription factor (Lubbers et al., 2019b). The FarA target genes required for the utilization of ferulic acid, p-coumaric acid and caffeic acid as well as fatty acids are *foxA* and *katA*. Based on homology with the FoxA protein of *A. nidulans* (Maggio-Hall and Keller, 2004), the *A. niger* FoxA (NRRL3_00672) protein was identified and considered to act as a candidate β-oxidation hydratase/dehydrogenase, and KatA (NRRL3_5990) is predicted to be a 3-keto-acyl CoA thiolase. Deletion of *foxA* or *katA* in *A. niger* results in reduced growth on hydroxycinnamic acids (ferulic acid, p-coumaric acid and caffeic acid) as well as on fatty acids (Lubbers et al., 2021a). In this study, we show that deletion of the FarA transcription factor leads to similar phenotypes indicating that the expression of *foxA* and *katA* s is probably controlled by FarA. The presence of putative FarA transcription factor binding sites (5’-CCTCGG) (van den Berg et al., 2010) in the promoters of *A. niger foxA* (at nucleotide position –225) and *katA* (at nucleotide position –400) is further in support that FarA is directly controlling their expression.

The first step in the oxidative peroxisomal degradation pathway of ferulic acid is catalyzed by hydroxycinnamate-CoA synthase encoded by *hcsA* (NRRL3_05989). Deletion of *hcsA* results in strongly reduced growth on several hydroxycinnamic acids but not on cinnamic acid (see above). However, growth on fatty acids was not affected (Lubbers et al., 2021a). Interestingly, also in the *hcsA* promoter a putative FarA binding site is present (at nucleotide position -871 before the start codon). Whether FarA is indeed directly binding to the promoter of these genes and required for the induced expression of ferulic acid requires further studies.

Based on our results, the FarB transcription factor in *A. niger* plays also a role in relation to degradation of aromatics and fatty acids. Its role in ferulic acid degradation seems less important than that of FarA, because growth of the *ΔfarB* strain is less affected than of the *ΔfarA* strain. An unexpected finding was the reduced growth of *ΔfarB* on benzoic acid and vanillic acid. The growth of the *ΔfarB* strain was as expected reduced on the short chain fatty acid valeric acid but not on long chain fatty acids. As in *A. flavus*, the FarB transcription factor in *A. niger* seems to be mostly involved in the regulation of enzymes involved in metabolism of short chain fatty acids (Luo et al., 2016).

In this study, we identified FarD as a new regulatory factor involved in the metabolism of hydroxycinnamic acids. Our results show that the absence of FarD reduces ability to grow on ferulic acid and other hydroxycinnamic acids. The *farD* gene was identified in a screen for mutants that were no longer able to induce the *faeB* gene, indicating that FarD, directly or indirectly, controls the induction of *faeB* in response to hydroxycinnamic acids. The mutant screen also yielded FarA loss-of-function mutants. The surprising finding is that the FarD protein lacks any known DNA binding motif, but contains the fungal specific transcription factor domain (PFAM04082) that is normally found in Zn(II)_2_Cys_6_ transcription factors. The fungal specific transcription factor domain is around 240 amino acids long and overlaps with the CDD (conserved domain database) cd12148 known as the fungal transcription factor homology domain (Fungal_TF_MHR). The cd12148 domain is generally around 400 amino acids in length. This domain is thought to play a role in regulating the transcriptional activity of these factors (Schjerling and Holmberg, 1996; MacPherson et al., 2006). To the best of our knowledge this is the first report that describes functional analysis of a gene encoding a protein with a transcription factor homology domain, but lacking the canonical Zn(II)_2_Cys_6_ function. The mechanism by which FarD exerts its function is unknown.

Besides a role in the induction of genes required for peroxisomal beta-oxidation, FarA has also been implicated in the induction of expression of genes encoding cutinases in *Fusarium solani, F. oxysporum, A. oryzae* and *A. nidulans* (Kämper et al., 1994; Li and Kolattukudy, 1997, Rocha et al., 2008, Garrido et al., 2012; Bermúdez-García et al., 2019). For *F. solani* it was shown that Ctf1 (FarA homolog) binds directly to the *cut1* promoter (Li and Kolattukudy, 1997). So far, a role for FarA (or any other Far protein) in directly inducing FaeB (or other feruroyl esterases) had not been described. The observation that in *farA* and *farD* mutants the *PfaeB-amdS and the PfaeB-lux* reporter gene are not induced in response to ferulic acid suggest that FarA (and/or FarD) are directly involved in the induction. A putative FarA binding site at position -474 upstream of the translational start site supports the possibility that FarA binds directly to its target effector genes.

The *amdS* selection method has been successfully used in several studies to identify regulatory mutants. Such a screen requires a gene which is specifically induced under a certain growth condition. The promoter of that gene is cloned in front of the *amdS* reporter gene which allows the selection of mutants that express the *amdS* gene under non-inducing conditions. Screens have been performed using genes specifically induced by galacturonic acid (exo-polygalacturonase X (*pgaX*)), arabinose (arabinofuranosidase A (*abfA*)) or ferulic acid (feruloyl esterase B (*faeB*)) respectively (Niu et al., 2017; Reijngoud et al., 2019; Reijngoud et al., 2021). In all these studies, the aim was to identify mutants resulting in constitutive expression of the target gene. In this study by using a counter selection strategy of *amdS*, we aimed to isolate mutants unable to induce *faeB* expression in order to find regulatory mutants. To screen for those mutants, we used the possibility to counterselect for the expression of *amdS* using fluoro-acetamide. In an initial experiment, it was confirmed that the induction of the *amdS* gene *via* the *faeB* promoter with ferulic acid resulted in lethality on fluoro-acetamide (**Figure 3**) which allowed the selection of mutants. To the best of our knowledge it is the first time that the a promoter-*amdS* reporter is used in a counter selection strategy to identify mutants defective in induction. Our results show that it is a powerful method to obtain and identify both gain- and loss of function mutations in trans-acting factors.

## Data availability statement

The datasets presented in this study can be found in online repositories. The names of the repository/repositories and accession number(s) can be found below: https://www.ncbi.nlm.nih.gov/, SRR18932555 https://www.ncbi.nlm.nih.gov/, SRR18932554 https://www.ncbi.nlm.nih.gov/, SRR18932553 https://www.ncbi.nlm.nih.gov/, SRR18932552.

## Author contributions

MA: Investigation, conceptualization, validation, data analysis, methodology, visualization. JR: Investigation, conceptualization, data analysis, methodology, data analysis. DT: Investigation, conceptualization, validation, data analysis, methodology, visualization, first draft manuscript writing. IR: DNA sequencing, data analysis, manuscript writing. YA: investigation, data analysis. HP: Conceptualization, data analysis, funding. NP: Conceptualization, data analysis, funding. JV: Conceptualization, data analysis, funding manuscript writing. PP: Conceptualization, data analysis, manuscript writing. AT: Conceptualization, data analysis, manuscript writing, funding. AR: Conceptualization, data analysis, original draft writing, funding acquisition, overall supervision. All authors agree to be accountable for the content of the work.

## Funding

This project was partially funded by the Genome Canada and Génome Québec and partially funded the Kluyver Centre for Genomics of Industrial Fermentation.

## Conflict of interest

Authors YA, HP, NP were employed by the company DSM Netherlands.

The remaining authors declare that the research was conducted in the absence of any commercial or financial relationships that could be construed as a potential conflict of interest.

## Publisher’s note

All claims expressed in this article are solely those of the authors and do not necessarily represent those of their affiliated organizations, or those of the publisher, the editors and the reviewers. Any product that may be evaluated in this article, or claim that may be made by its manufacturer, is not guaranteed or endorsed by the publisher.
