# Supplementary material for: Utilization of ferulic acid in Aspergillus niger requires the transcription factor FarA and a newly identified Far-like protein (FarD) that lacks the canonical Zn(II)2Cys6 domain
Source: Front Fungal Biol. 2022 Nov 8;3:978845. doi: 10.3389/ffunb.2022.978845 (PMC10512302; doi:10.3389/ffunb.2022.978845)
Supplement: Supplementary file 1 [file Presentation_1.pptx]

## Slide 1
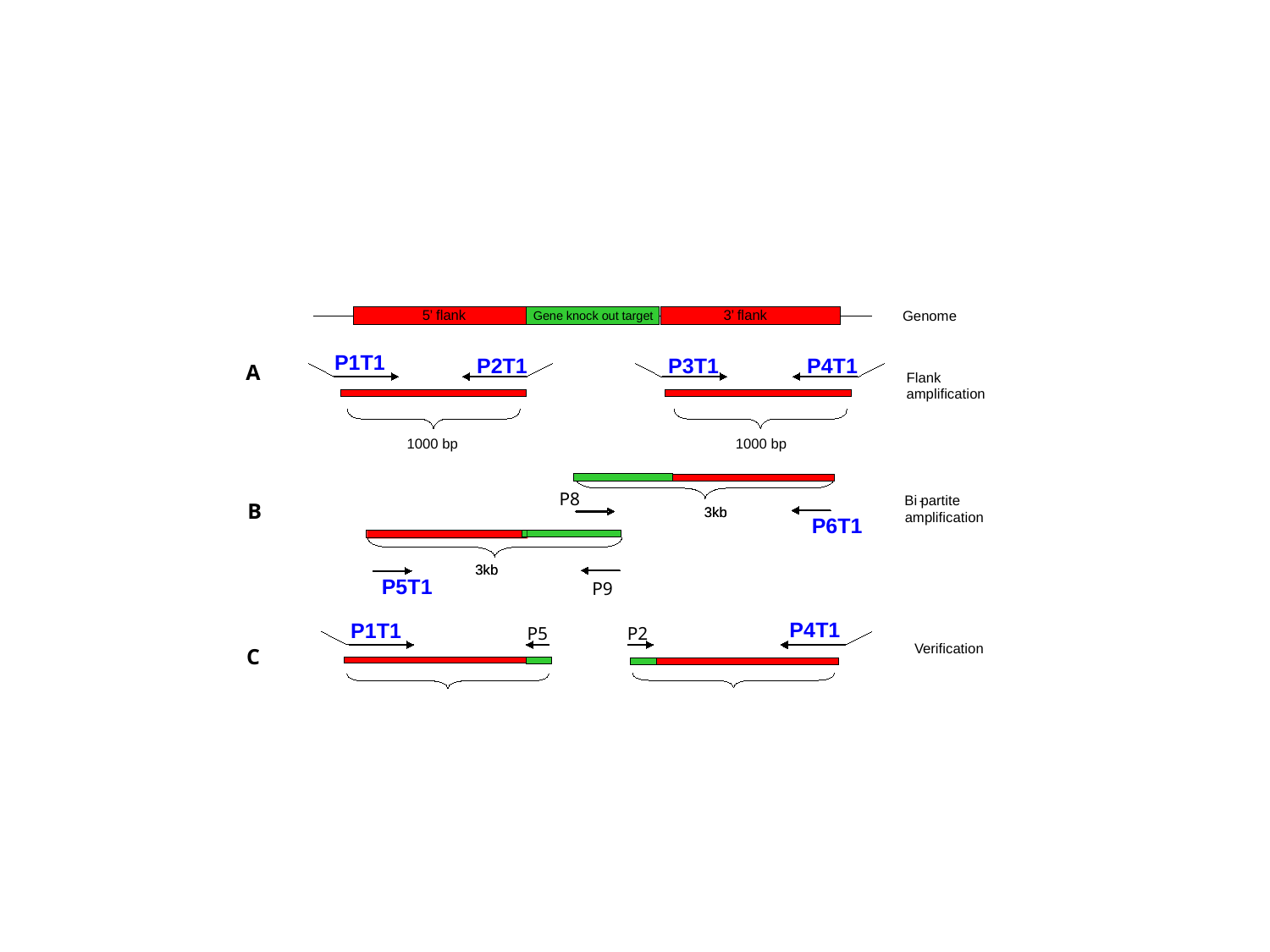

5
5
’
’
flank
flank
3
’
flank
Genome
Gene knock out target
3
’
flank
P1T1
P2T1
P3T1
P4T1
A
Flank
amplification
1000 bp
1000 bp
P8
Bi
-
partite
B
3kb
3kb
amplification
P6T1
3kb
3kb
P9
P5T1
P5
P2
P4T1
P1T1
Verification
C
