# Supplementary material for: Utilization of ferulic acid in Aspergillus niger requires the transcription factor FarA and a newly identified Far-like protein (FarD) that lacks the canonical Zn(II)2Cys6 domain
Source: Front Fungal Biol. 2022 Nov 8;3:978845. doi: 10.3389/ffunb.2022.978845 (PMC10512302; doi:10.3389/ffunb.2022.978845)
Supplement: Supplementary file 2 [file Presentation_2.pptx]

## Slide 1
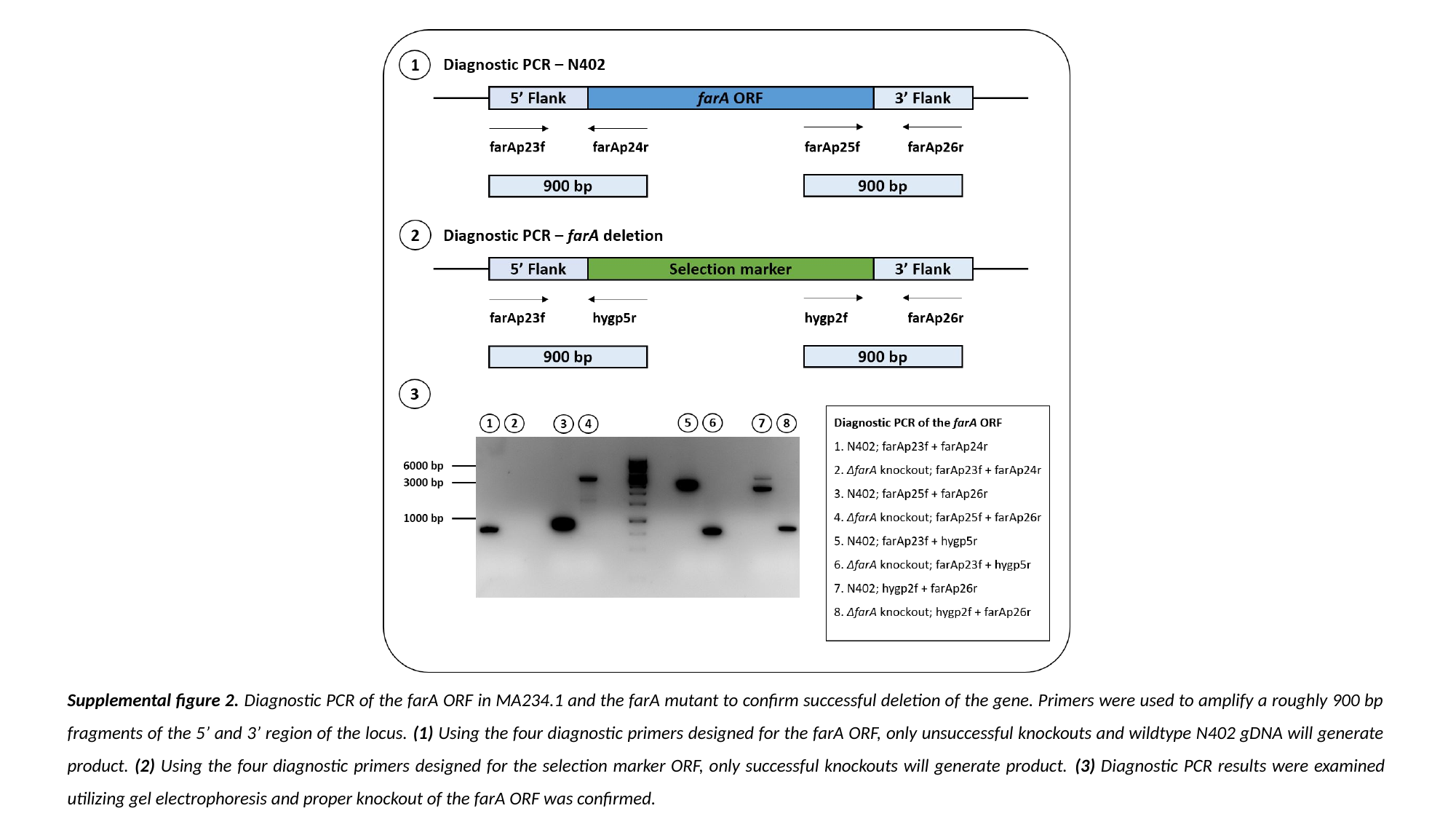

Supplemental figure 2. Diagnostic PCR of the farA ORF in MA234.1 and the farA mutant to confirm successful deletion of the gene. Primers were used to amplify a roughly 900 bp fragments of the 5’ and 3’ region of the locus. (1) Using the four diagnostic primers designed for the farA ORF, only unsuccessful knockouts and wildtype N402 gDNA will generate product. (2) Using the four diagnostic primers designed for the selection marker ORF, only successful knockouts will generate product. (3) Diagnostic PCR results were examined utilizing gel electrophoresis and proper knockout of the farA ORF was confirmed.
