# Supplementary figures and images for: Utilization of ferulic acid in Aspergillus niger requires the transcription factor FarA and a newly identified Far-like protein (FarD) that lacks the canonical Zn(II)2Cys6 domain
Source: Front Fungal Biol. 2022 Nov 8;3:978845. doi: 10.3389/ffunb.2022.978845 (PMC10512302; doi:10.3389/ffunb.2022.978845)

## Slide 1
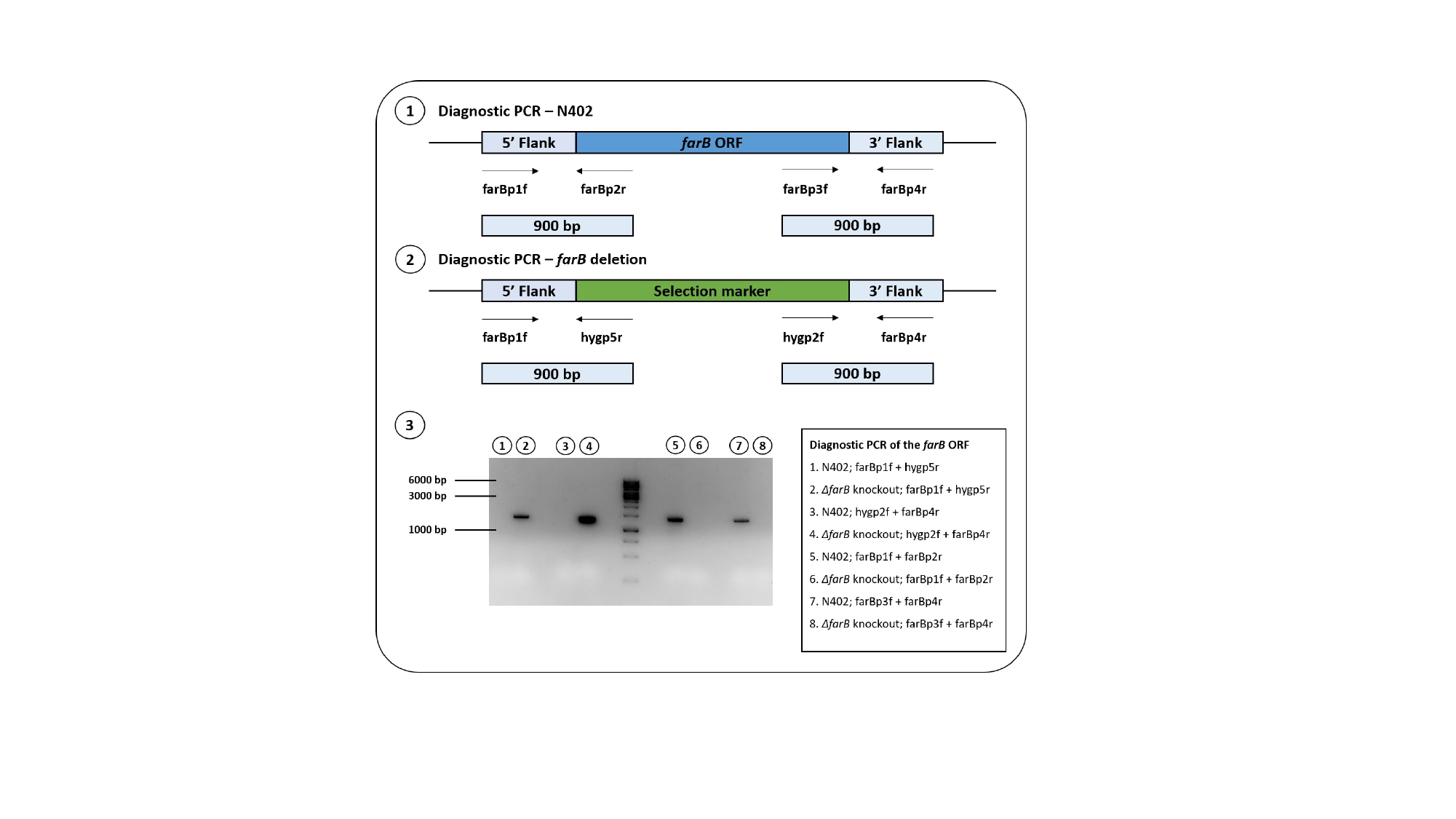

Supplement: Supplementary file 3 [file Presentation_3.pptx]

## Slide 1
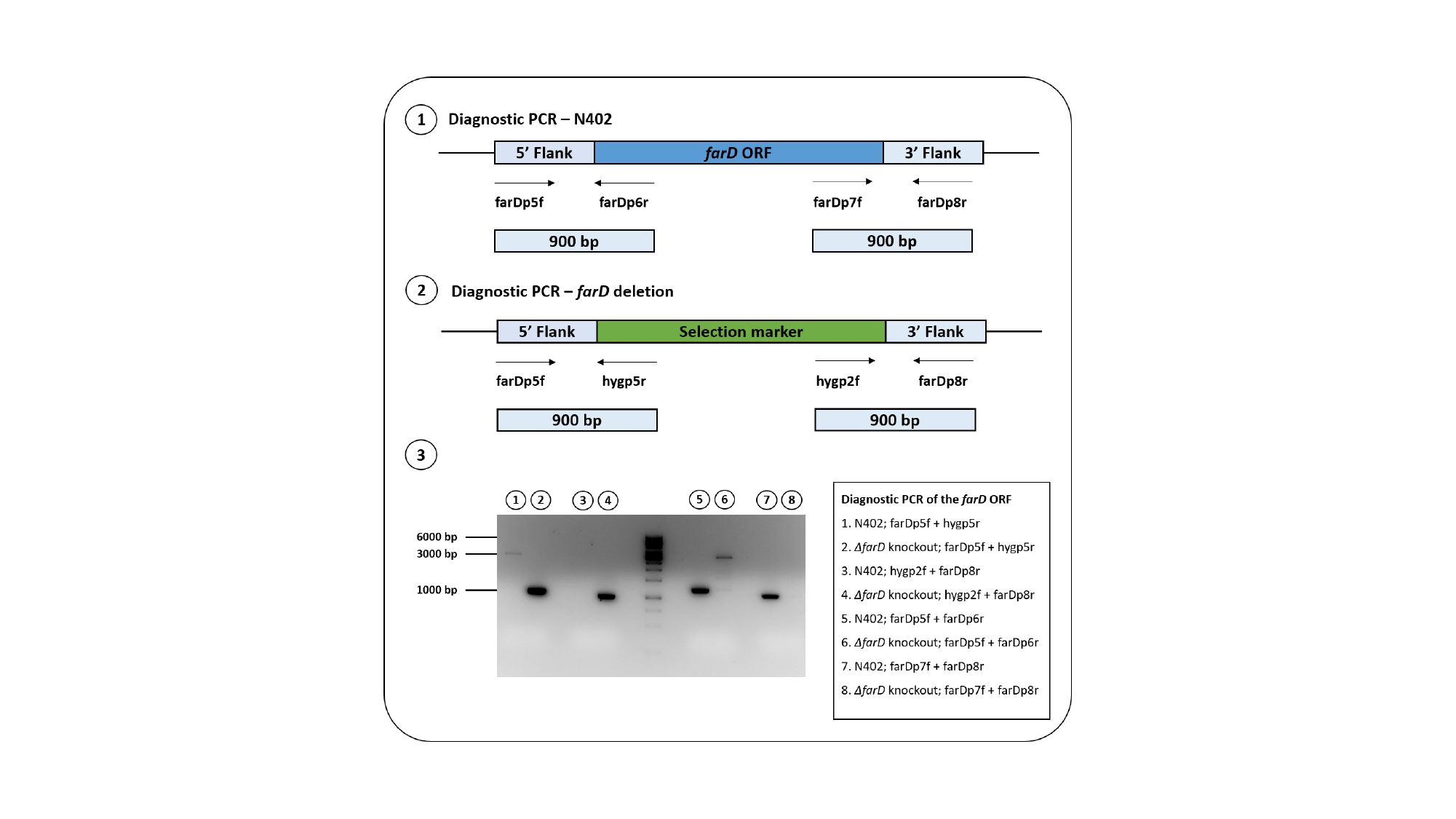

Supplement: Supplementary file 4 [file Presentation_4.pptx]
