# Supplementary material for: Utilization of ferulic acid in Aspergillus niger requires the transcription factor FarA and a newly identified Far-like protein (FarD) that lacks the canonical Zn(II)2Cys6 domain
Source: Front Fungal Biol. 2022 Nov 8;3:978845. doi: 10.3389/ffunb.2022.978845 (PMC10512302; doi:10.3389/ffunb.2022.978845)
Supplement: Supplementary file 5 [file Table_1.docx]

**Supplementary Table 1.** Primers used in this study

| **Primer name** | **Sequence (5’ to 3’)** | **Used for** | **Remarks** |
| --- | --- | --- | --- |
| hygP2f | CATGCATGGTTGCCTAGTGAA | Diagnostic PCR TF deletion strains and complementation | - |
| hygP5r | ATCCACTGCACCTCAGAGCC | Diagnostic PCR TF deletion strains and complementation | - |
| hygP8f | AAAGTTCGACAGCGTCTCC | Fusion PCR for 3’ flank *hygB* split marker deletion | - |
| hygP9r | GGCGTCGGTTTCCACTATC | Fusion PCR for 5’ flank *hygB* split marker deletion | - |
| phleoP6f | AAGTTGACCAGTGCCGTTCC | Fusion PCR for 3’ flank *phleo* split marker deletion | - |
| phleoP8r | GGAAGTTCGTGGACACGACC | Fusion PCR for 5’ flank *phleo* split marker deletion | - |
| pTE1for | CCTTAATTAAACTCCGCCGAACGTACTG | gRNA flank amplification including GOI target | - |
| pTE1rev | CCTTAATTAAAAAAGCAAAAAAGGAAGGTACAAAAAAGC | gRNA flank amplification including GOI target | - |
| farAP19f | CGAAACTCTCTCACCCTCGC | Amplifying *farA* 5’ flank for split marker deletion | - |
| farAP20r | CAATTCCAGCAGCGGCTTGGGGAGGAAACG ACTCGAGGGT | Amplifying *farA* 5’ flank for split marker deletion | Split marker overlap underlined |
| farAP21f | ACACGGCACAATTATCCATCGTGACATGGC ATGGGTGGTTGTTTGA | Amplifying *farA* 3’ flank for split marker deletion | Split marker overlap underlined |
| farAP22r | CCTTCTTCACCGCGGCATCACT | Amplifying *farA* 3’ flank for split marker deletion | - |
| farAP23f | GCTGCTGCTGCTTGCTTTTG GG | Diagnostic PCR of 5’ *farA* deletion | - |
| farAP24r | CCCGTGCGCACAGTAATACC | Diagnostic PCR of 5’ *farA* deletion | - |
| farAP25f | AAGTTGATTCCGGCGGCT | Diagnostic PCR of 3’ *farA* deletion | - |
| farAP26r | CCTTCTTCACCGCGGCATCA CT | Diagnostic PCR of 3’ *farA* deletion | - |
| farAP27f | GCGGCCGCCAGCTATCCAACCATCTAGACT | Amplifying *farA* gene for complementation | *NotI* site underlined |
| farAp28r | GGCGCGCCGATTGGACAAAAGAGAGATGAC | Amplifying *farA* gene for complementation | *AscI* site underlined |
| farAP29f | CTCATTGCCTCTTTCTCTCTC | Sequencing *farA* | - |
| farAP30f | GTTTCAGAAGGTGAGTGTTGAC | Sequencing *farA* | - |
| farAP31f | CAAGAAGAACCAAACCAAGTC | Sequencing *farA* | - |
| farAP32f | CCATCTTTACTTCTTCTCCAAG | Sequencing *farA* | - |
| farAP33f | GGTACACGTACAGTTCTTCCTT | Sequencing *farA* | - |
| farAP34f | CTATTTGAATCCATTCTCGG | Sequencing *farA* | - |
| farAP35f | GTGTTTGGTATCGAAAGTCAG | Sequencing *farA* | - |
| FarBP1f | GCTGAGGAACTGGGTGGAAAC | Diagnostic PCR of 5’ *farB* deletion | - |
| FarBP2r | CCTCTTGGATTTGGCCTCG | Diagnostic PCR of 5’ *farB* deletion | - |
| FarBP3f | GACATGAACACTTTCGCAGCC | Diagnostic PCR of 3’ *farB* deletion | - |
| FarBP4r | ATTTCCTTGGCGGCAGGT | Diagnostic PCR of 3’ *farB* deletion | - |
| FarBP5f | CGTTGAAGTCTCCAGTGGG | Diagnostic PCR of 5’ *farB* deletion | Backup primer KIT mutants |
| FarBP6r | CTGTGGCTGTGAGTAAGATG | Diagnostic PCR of 5’ *farB* deletion | Backup primer KIT mutants |
| FarBP7f | TCAACTAAAATCCCCCGGCGCG | Amplifying *farB* 5’ flank for split marker deletion | - |
| FarBP8r | CAATTCCAGCAGCGGCTTCATCCGTGAGTAGGCCAGCCTT | Amplifying *farB* 5’ flank for split marker deletion | Split marker overlap underlined |
| FarBP9f | ACACGGCACAATTATCCATCGGCTTTCTGCATTGCCCTTGGCG | Amplifying *farB* 3’ flank for split marker deletion | Split marker overlap underlined |
| FarBP10r | GGGACTGGTATGCACGGGACCT | Amplifying *farB* 3’ flank for split marker deletion | - |
| farDP1f | CGCTGGTCAGGTAATTCAGGCCC | Amplifying *farD* 5’ flank for split marker deletion | - |
| farDP2r | CAATTCCAGCAGCGGCTTGCAGCTGCGCAG AAGATCACA | Amplifying *farD* 5’ flank for split marker deletion | Split marker overlap underlined |
| farDP3f | ACACGGCACAATTATCCATCGAGGGAGCTG AGGTGGTTGTTCGT | Amplifying *farD* 3’ flank for split marker deletion | Split marker overlap underlined |
| farDP4r | CACGAACAAATCCAACGGCGCG | Amplifying *farD* 3’ flank for split marker deletion | - |
| farDP5f | GATTCTCGCCCGTTCGTTCGCA | Diagnostic PCR of 5’ *farD* deletion | - |
| farDP6r | CAGCTCCGAGAAGGTCTGGA | Diagnostic PCR of 5’ *farD* deletion | - |
| farDP7f | GGGCAGAGGTCCATGAGTCA | Diagnostic PCR of 3’ *farD* deletion | - |
| farDP8r | TACACCGGTGTCCGCCAGGATT | Diagnostic PCR of 3’ *farD* deletion | - |
| farDP9f | ATTATACTGTGTGCTTCATGGG | Amplifying *farD* 5’ flank for split marker deletion | Backup |
| farDP10r | CAATTCCAGCAGCGGCTAGATCACATTCAGCATAAGAGG | Amplifying *farD* 5’ flank for split marker deletion | Backup, split marker overlap underlined |
| farDP11f | GCGGCCGCCTGACGGAAGAAGTATGTAAAC | Amplifying *farD* gene for complementation | *NotI* site underlined |
| farDP12r | GGCGCGCCGTACAAAGTGTCAAGAAGAAGC | Amplifying *farD* gene for complementation | *AscI* site underlined |
| farDP13f | GTACTTAAGAGGAACGACACCA | Sequencing *farD* | - |
| farDP14f | CTCTTATTGCTCTCCTCGAC | Sequencing *farD* | - |
| farDP15f | GAATTTGGTGGAGCTTATACTC | Sequencing *farD* | - |
| farDP16f | GTGTAACCGTTATCATCCCAG | Sequencing *farD* | - |
| farDP17f | TTTGACTTTGATCTCGACCT | Sequencing *farD* | - |
