# Supplementary material for: Utilization of ferulic acid in Aspergillus niger requires the transcription factor FarA and a newly identified Far-like protein (FarD) that lacks the canonical Zn(II)2Cys6 domain
Source: Front Fungal Biol. 2022 Nov 8;3:978845. doi: 10.3389/ffunb.2022.978845 (PMC10512302; doi:10.3389/ffunb.2022.978845)
Supplement: Supplementary file 8 [file Table_4.docx]

>jgi|Aspni_NRRL3_1|06630|FarB|874aa

MTNTAVSTTSPTPQSTVETEAKSKRKASTAGLPANARPVKRRASKACCCCRARKVRCDVVENGSPCTNCRLDQVECIVTESKRRKKSRVEVENANPHQLSQSPAEIPDDGASLFRRLSECHGLSDMAPASPSQRSVDLDQGQHMPHLLYQSQANRIGSGDRFRRRMAPNPAVPATLPLHHVTSQIQQLLDPSFANARSGGVVLPDYIRGLPPRLQKEDIDYLSMKGALTVPDVGLRNELLKSYIHYVHTYMPLLDLEDFLQTIVQNDGIRRMSLLLFQAVMFAGTAFIDLKHLHAAGYPTRKAARKAFFQRARLLYDFDYEVDRISLVQSLLLMTYWYETPDDQKDTWHWMGVSLSLAHTIGLHRDPGNSRMDVRRQRMWKRIWWSTYTRDRLIALGMRRPMRVKDDDCDVPMLTLDDFEFHPFSPEIVSMVGNSEILQSVSHQRELALMFIEKAKLCLCVSHVLSAQYSVLSHKFGGTMETTMMLVPKKSAAETFEVRRCDQELEDWLAHLPTEIRYAPAAPSKLNEAQEVLHSHRALLKMVYLTTSSALHRPQVLPAIPFPSMDAELQEISRNKVRFAAIEITNIAQDLHSLDLTRYFPTTGVTVLLPAVIIHLLDIKSSDHNVRMTSLHRFYQCMRILQRLREIYASADFATSFLEAAIRKAGIQLTVAPQDVQARTNNIVDTSARVNTLTPPPDSLAQKIPDLTYPKSDGARRASQLPKESGPEFASTPPPSDGSENGSTSNINPSYHQDAFTIPNFDSELSISELMDLANDAEVTQNDFDALINFDDAGADFLAPDDGLNTGNGNGKNYGFGLGTMNNLSDIMGFDAEKQGVDLTGLGDGQLADDRAAASRNDMNTFAADLDAELGLSL

>jgi|Aspca4|1107189|FarB|875aa

MTSTAVSTTTPTPQTAVDMESKTKRKASTAGLPANARPVKRRASKACCCCRARKVRCDVVENGSPCTNCRLDQVECIVTESKRRKKSRVEVDNANPQQLSQSPAEVPDDGASLFRRLSECHGLSDMIPGSPSQRSVDLDQGQHIPHLLYQSQASRIGSGDRFRRRMAPNPAVPATLPLHHVTSQIQQLLDPSYANARSGGVVLPDYIRGLPPRLQKEDIDYLSMKGALTVPDAGLRNELLKSYIHYVHTYMPLLDLEEFLQTIVQNDGIRRLSLLLFQAVMFAGTAFIDLKHLHAAGYPTRKAARKAFFQRARLLYDFDYEVDRISLVQSLLLMTYWYETPDDQKDTWHWMGVSLSLAHTIGLHRDPGNSRMDLRRQRMWKRIWWSTYTRDRLIALGMRRPMRVKDDDCDVPMLTLDDFEFHPFSPEIVNMVGNSEILQSVSHQRELALMFIEKAKLCLCVSHVLSAQYSVLSHKFGGTMETTMMLVPKKSAAETFEVRRCDQELEDWLAHLPAEIQYAPAAPSKLSEAQEVLHSHRALLKMVYLTTSSALHRPQVLPAIPFPSMDAELQEISRNKVRFAAIEITNIAQDLHSLDLTRYFPTTGVTVLLPAVIIHLLDIKSSDPNIRMTSLHRFYQCMRILQRLREIYASADFATSFLEAAIRKAGIQLTVAPQDVHPRTNSTLDTTARVNTLTPPPDSLAQKIPGLTYPKSDGAGLTGQLTKGSGPGFASTPPPSDGSENGSTNNLNPSYHQDAFPIPNFDSELSISELMGLANDAEVTQNDFDALINFDDAGADFLAADDGLNPNGNASGKSYGFGLGPVGDLSDLMGFDAENKGGDLTGLGDGQLVENRVAAPRADVASLAADLDAELGLSL

>jgi|Asfl2_3|2237094|873aa

MTNLTASPSSSNMAENEAKGKRKASTAGLPANARPVKRRASKACCCCRARKVRCDVVENGSPCTNCRLDQVDCIVTESKRRKKSRVEVDNPNHQLSQSPAEAPEDGSLLRRLSECHGLSDVAPASPSQRSVDLDQGQHMPHLLYQSQVSRIGAGPERYRRRMAPNPAVPATMPLHHVTSQIQQLLDPSFANARSGGIILPDYIRGLPPRLQKEDIDYLAMKGALTVPDVGLRNELLKAYIHYVHTYMPLLDLEDFLQTIAQNDGIRRMSLLLFQAVMFAGTAFVDLKHLQAAGYSSRKAARKSFFQRARLLYDFDYEVDRISLVQSLLLMTYWYETPDDQKDTWHWMGVSLSLAHTIGLHRDPGNSRMDVRRQRMWKRIWWSTYTRDRLIALGMRRPMRVKDDDCDVPMLTLDDFEFHPFSPEIVSMVGNSEVLQNVSHQKELALMFIEKAKLCLCVSHVLSAQYSVLSHKFGGTMETTMMLVPKKSAAETFEVRRCDQELEDWLAHLPSEIQYAPMAPAKLTEAQEVLHSHRALLKMVYLTTSSALHRPQVLPAMPFPSTDAELQDISRNKVRFAAVEITNIAQDLHALDLTRYFPTTGVTVLLPAVIIHLLDIKSSDPNVRMVSLQRFYQCMRILQRLREIYASADFATSFLEAAIRKAGIQLTVAPQDVQSRNNCTFDSVRLNTLTPPPDSLAQKIPDLTYPKTSGTRLAGEAAEASGFASTPPPSDGSENGSTNNINPHYHQDAFAIPNLDSDLSISELMDLANDAEVTQNDFDALINFDDTGAELFAADDGLDLNGNPKGQGYGFNIGTMDNVPDLFGTESKGVGLTGLGNGQLHEDRTSTTLGANEAPRATELDGIADLEAELGLNL*

>jgi|Aspte1|25|FarB|843aa

MSDLATSSTPTAAPTNSVDSDSKGKRKASTAGLSANARPVKRRASKACCCCRARKVRCDV

VENGSPCTNCRLDQVECIVTESKRRKKSRVEVDNSHRQLSQSPADLSEDGGNLFRRLSEC

NGMPDVAPASPSQRSVDLDQGQHMPHLLYQSQVNRIGAGEQFRRRMAPNPAVRATMPLQN

VTSQIQQLLDPTFANVRSSGVVLPDYIRGLPQRLQKEDIEYLAMKGALTVPDVGLRNELL

KAYIHYVHTYMPLLDLEDFLQTIVQNDGIRRMSLLLFQAVMFAGTAFVDLKHLHAAGYSS

RKAARKAFFQRARLLYDFDYEVDRISLVQSLLLMTYWYETPDDQKDTWHWMGVSLSLAHT

IGLHRDPGNSRMDARRQRMWKRIWWSTYTRDRLIALGMRRPMRVKDDDCDVPMLTLDDFE

FHPFSPEIVSMVGNSEILQDVSHQKELALMFIEKAKLCLCVSHVLSAQYSVLSHKFGGTM

ETTMMLVPKKSAAETFEVRRCDQELEDWLAHLPAEIQYAPAAPAKLSEAQEVLHSHRALL

KMVYLTTSSALHRPQVLPAIPFPSTDTELQEISRNKVRFAAVEITNIAQDLHSLDLTRFF

PTTGVTVLLPAVIIHLLDIKSSDPNVRMTSLQRFYQCMRILQRLREIYASADFATSFLEA

AIRKAGIQLTVAPNDVHTRAGASTTDGGASRVNTLTPPPDSLAQKIPDLTYPKPEGNGVA

GSSAQEPLFASTPPPSDGSENGSTNNINPSYAQDAFSIPNLDADLSLSELMDLANDAEVT

QNDFDALINFDDAGADFFTADDGANGKGLGFGMMHNVPDMGDLATEAPRAEVDLDAELGL

NLN

>jgi|Aspfu1|9463|FarB|885aa

MTDLAVSSTPASANTMESESQSKRKASAAGLSANSRPVKRRASKACCCCRARKVRCDVVENGSPCTNCRLDQVECVVTESKRRKKSRIEVENGTRQPSQSPADGLDDAGSFLRRLSESHGLNADIAPESPSQQSVDLDQGHHMPHLLYQSQANRIGGGNHFRRRMAPNPAVPATLPLHHVTSQIQQLLDPSFSNARSGGVLLPDYIRGLPSRLQREDIEYLSMKGALTVPDVGLRNELLKAYIHYVHTYMPLLDLEEFLQTIVQNDGIHRMSLLLFQAVMFAGTAFIDLKHLHAAGYPSRKAARKVFFQRARLLYDFDYEVDRISLVQSLLLMTYWYETPDDQKDTWHWMGVSLSLAHTIGLHRDPGNSRMDARRQRMWKRIWWSTYTRDRLIALGMRRPMRVKDDDCDVPMLTLDDFEFHPFSPEIVRMVGNSEILQNVEHQKKLALMFIEKAKLCLCVSHVLSAQYSVLSHKFGGTMETTMMLVPKKSAAETFEVRRCDQELEDWLANLPAEIQYTPAASAKLTEAQEVLHSHRALLKMVYLTTSSALHRPQVLPANPFPSMDAELQEISRNKVRFAAVEITNIAQDLHSLDLTRYFPTTGVTVLLPAVIIHLLDIKSSDPSVRMTSLQRFYQCMRILQRLREIYASADFATSFLEAAIRKAGIQLTVAPQDVQARPGSSFDAGARVDTLTPPPDSLAQKIPDLTYPKPAVVGTARQPVEEGDTLFASTPPPSDGSENGSTANINPNYHRDAFSIPNLDSELSISELMDLANDAEVTQNDFDALINFDDAGADFFTADDHLNLNGSGPNSKGFGFSMDPINGMSDLMGLDAGNKPDIASLGDRQLHSDHMATTTGHSNTGTAASESNGLTANVDADLGICLSG*

>jgi|Aspor1|11322|FarB|873aa

MTNLTASPSSSNMAENEAKGKRKASTAGLPANARPVKRRASKACCCCRARKVRCDVVENGSPCTNCRLDQVDCIVTESKRRKKSRVEVDNPNHQLSQSPAEAPEDGSLLRRLSECHGLSDVAPASPSQRSVDLDQGQHMPHLLYQSQVSRIGAGPERYRRRMAPNPAVPATMPLHHVTSQIQQLLDPSFANARSGGIILPDYIRGLPPRLQKEDIDYLAMKGALTVPDVGLRNELLKAYIHYVHTYMPLLDLEDFLQTIAQNDGIRRMSLLLFQAVMFAGTAFVDLKHLQAAGYSSRKAARKSFFQRARLLYDFDYEVDRISLVQSLLLMTYWYETPDDQKDTWHWMGVSLSLAHTIGLHRDPGNSRMDVRRQRMWKRIWWSTYTRDRLIALGMRRPMRVKDDDCDVPMLTLDDFEFHPFSPEIVSMVGNSEVLQNVSHQKELALMFIEKAKLCLCVSHVLSAQYSVLSHKFGGTMETTMMLVPKKSAAETFEVRRCDQELEDWLAHLPSEIQYAPMAPAKLTEAQEVLHSHRALLKMVYLTTSSALHRPQVLPAMPFPSTDAELQDISRNKVRFAAVEITNIAQDLHALDLTRYFPTTGVTVLLPAVIIHLLDIKSSDPNVRMVSLQRFYQCMRILQRLREIYASADFATSFLEAAIRKAGIQLTVAPQDVQSRNNCTFDSVRLNTLTPPPDSLAQKIPDLTYPKTSGTRLAGEAAEASGFASTPPPSDGSENGSTNNINPHYHQDAFAIPNLDSDLSISELMDLANDAEVTQNDFDALINFDDTGAELFAADDGLDLNGNPKGQGYGFNIGTMDNVPDLFGTESKGVGLTGLGNGQLHEDRTSTTLGANEAPRATELDGIADLEAELGLNL*

>jgi|Aspgl1|163665|FarB|883aa

MTNATTATTTPSHPKNTMSSTDSQAKRKASTAGMSVNTRPVKRRASKACCCCRARKVRCDVVENGSPCTNCRLDQVECVVTESKRRKKSRVEAENANNVSDSPPDASEDSGNFFRRFSESRRLSDVAPASPSQHSLDLDQGSHMPHLLYQNQAERIGAESRFGRGMAPNPAVPATLPLHHVTSQIQQLLDPSFASPRSGGVVLPDYIRGLPARLQKEDIDYLAMKGALTIPDVNLRNELIKSYIHYVHTYMPLLDIEDFLQTIIQNDGINRMSLLLFQAVMFAGTAFIDLKHLHGAGYTSRKAARKAFFQRARLLYDFDYEVDRISLVQSLLLMTYWYETPDDQKDTWHWMGVSLSLAHTIGLHRDPGNSRMDLRRQRMWKRIWWSTYTRDRLIALGMRRPMRVKDDDCDVPMLTLDDFDFHPFSPEIVAMVGNSEVLHNIEHQRELALMFIEKAKLCLCVSHVLSAQYSVLSHKFGGTMETTMMLVPKKSAAETFEVRRCDQELEDWLANLPPEIQYSPAASLKLTEAQEVLHSHRALLKMVYLTTSSALHRPQVLPSIPFPSMDAELQDISRNKVRYAAVEITNIAQDLHSLDLTRYFPTTGVTVLLPAVIIHLLDIKSSDPNVRMTSLQRFYQCMRILQRLREIYASADFATSFLEAAIRKAGIQLTVAPQDVQQPPPVNADTASSTMPDPTRLNTLTPPPDSLAQKIPDLTYPKSNDTGALGAAEEGGPLFVSTPPPSDGSENGSTNNLNPKFPPSGAFQIPNMESELSLSQLMDLANDAEVTQNDFDALINFDDAGADFFAAENNSNNTTAINTTTNNNTNDHESHDGNDFVDHIPDVMNLDAGDKGVNFPGLADAVEAPRSEAQGITADLDADLGLF*

>jgi|Aspcl|28|FarB|880aa

MANLAVSSSTATPANTADTESQGKRKASTAGLSANSRPVKRRASKACCCCRARKVRCDVVENGSPCTNCRLDQVECVVTESKRRKKSRVEVENANRQPSQSPVDVSEDSASFLRRLSECHELPPDLAPGSPSQNSIDLDQGQHMPHLLYQNQASRIGGGDQFRRRMAPNPPPTLPLHHVTSQIQQLLDPSFSNSRSAGVLLPDYIRGLPSRLQKEDIEYLAMKGALTVPDVGLRNELLKVYIHYVHTYMPLLDLEEFLQTIVQNDGIHRMSLLLFQAVMFASTAFIDLKHLQAAGYPTRKAARKVFFQRARLLYDFDYEVDRISLVQSLLLMTYWYETPDDQKDTWHWMGVSLSLAHTIGLHRDPGNSRMGARRQRMWKRIWWSTYTRDRLIALGMRRPMRVKDDDCDVPMLTLDDFEFHPFSPEIVSMIGDSEILQNVDYQKELALMFIEKAKLCLCVSHVLSAQYSVLSHKFGGTMETTMMLVPKKSSAETFEVRSCDQELEDWLANLPAEIQYSPAAPAKLTEAQEVLHSHRALLKMVYLTTSSALHRPQVLPASPFPSMDAELQEMSRNKVRFAAIEITNIAQDLHSLDLTRYFPTTGVTVLLPAVIIHLLDIKSSDPSVRMTSLQRFYQCMRILQRLREIYASADFATSFLEAAIRKAGIQLTVAPQDVQARSTASFDAPTRVNTLTPPPDSLAQKIPDLTYPKPAASVRIARPSVGGSETLFASTPPPSDGSENGSTSNINPSYHPNAFSIPNLDSDLSISELMDLANDAEVTQNDFDALINFDDAGADFFSADDHMNLNGAGSNHSKGYGFGMDSMPGMSDVMGFDHESKTEMSTRGGGHGSAALRHNTTETVVGASSLTTSLDAELGLGLSV*

>jgi|Aspnid|7061|FarB|853aa

MTDSPVAPNPAVETDPKSKRKASSAGLSANSRPVKRRASKACCCCRARKVRCDVVENGSPCTNCRLDQVECVVTESKRRKKSRVDTEISNPQLSQSPAEILDDGALFGGLGDTQGIPHVAPTSPSQGSVDMEQGQHMPHLLYQSQVNRVDPGDRFRKRMAPNPLVPSSMPLSHVTSEIQQLLDPSFGSPRSSGIVLPDYIRGLPARLQKEDIDYLAMKGALTVPDVTLRNELLKAYIHYVHTYMPLLDLEDFLQTIVQNDGIRRMSLLLFQAVMFAGTAFIDLKHLQAAGYPSRKSARKSFFQRARLLYDFDYEVDRISLVQSLLLMTYWYETPDDQKDTWHWMGVSLSLAHTIGLHRDPANSRMDVRRQRMWKRIWWSTYTRDRLIALGMRRPMRVKDDDCDVPMLTLDDFEFHPFSPEIVSMVGNSEILQSVAHQRELASMFIEKAKLCLCVSHVLSAQYSVLSHKFGGTMETTMMLVPKKSAAETFEVRRCDQELEDWLAHLPSEIQYAPAAPAKLSEAQEVLHSHRALLKMVYLTTSSALHRPQVLPAVPFPSMDTELQDMSRNKVRFAAIEITNIAQDLHSLDLTRYFPTTGVTVLLPAVIIHLLDIKSSDQNVRMTSLQRFYQCMRILQRLREIYASADFATSFLEAAIRKAGIQLTVPPQELQKRSNNSGASRNTTLTPPPDSLAQKIPDLTYPKTGSMGGMTLNLVDEAQPAFASTPPPSDGSENGSTNNINPNYHRDAFRIPNIDDTEMSLSQLMDLANDAEVTQNDFDALINFDDAGADFFSSENGVENMTGDGNSKNFPFTFQDMVGFDSGNKSDAAASNGADAAQTEAPNLDMDLGLSLNA*

>jgi|Aspzo1|133335|FarB|885aa

MATTTATATATTADSDSQSKRKASTAGLSANSRPVKRRASKACCCCRARKVRCDVVENGSPCTNCRLDQVECVVTESKRRKKSRVDVENTNNSADSPIEAPDEPHVHFRRPSETHGLLEGIPESPSQRSVDLDAGHHMPHLLYQKQVNRIGAEDRYRRRMAPNPAVPETLPMQNMTAQIRQLLEPSFSPASGFLPDYIRGLPPRLQKEDIDYLATKGALTVPDLTLRNELLKSYIHYVHTYMPLLDLEEFLQTIARNDGIHRMSLLLFQAVMFAGTAFIDLKHLLAAGYPNRKAARKVFFQRARLLYDFDYEVDRISLVQSLLLMTYWYETPDDQKDTWHWMGVSLSLAHTIGLHRDPGNSRMDLRRQRMWKRIWWSTYTRDRLIALGMRRPMRVKDDDCDVPMLTLDDFEFHPFPPEIVSMVGDSEILQNVNHQRELALMFIEKAKLCLCVSHVLSAQYSVLSHKFGGTMETTMMLVPKKSAAETFEVRRCDQELESWLANLPAEIQYSPSMTRKLPEAQEVLHSHRALLKMIYLTTSSALHRPQVLPASPYPSTDAELQELSRGKVRFAAIEITNIAQDLHVLDLTRFFPTTGVTVLLPAVIIHLLDIKSSDPNVRMTSLQRFYQCMRILQRLREIYASADFATSFLEAAIRKAGIQLTVAPQEVQRLNGNSNSSSSSSNTVSDGTLRMHTLTPPPESLAQKLPDLTYPKPPSARTAPRFIGGDLERGLPFASTPPDSDSSENGSTQNINPNYMPDTFTMQNMVNPDHLSLTELMDLANDAEVTQNDFDALINFDDAGADFFAADDGLNLDPTLDASSSSSSSLEKPAATATAYGFDIGVDLGLDADKTLDFHGLSKTSTSTNHPSAEKSMALIAEVEKELAF*

>jgi|Talst_2|12626|FarB|876aa

MASTTSSPPPETASSQNKRKASAAGLSAGNRPIKRRASRACCCCRARKVRCDVVENGSPCTNCRLDQVECVVTESRRRKKFRVETEAVKTTPDSSTELSEDAAKRRTSSHGLDGLDQSGLDGLTKDDPLSPFQESIDLDQGQHVPHFIYQSRGSHIDNENLPERRSNYSQSLSIAANIGPSFADAIHQLLTPPSQSNQLPDYIRRLPQRLQTEDLNYLQAKGALTIPDRALRDALLKAYIHYVHPYMPLLDLEEFLQILARNDGTHHVSLLLFQAVMFAGTAFVEMEALRNAGYNSRKTARKVFFQRARLLYDFDYEVDRISLVQSLLLMTYWYETPDDQKDTWHWMGVSLSLAHTIGLHRDPSNSGMGPRRKQLWKRIWWSTYTRDRLIALGMRRPMRVKDDDCDVPMLTLDDFEFKRFPPEVLNMLGDCELLQNTEHQRHLAMMFIEKAKLCLCVSHVLSAQYSVLSHRFGGTTETTMMLVPKKSEAGTIQVQKCDEELEKWQANLPSEIRYTSLEGVDSLSPAESSLQVHRALLRMIYLTTSSALHRPQVLPAMPFPAVEAALQEISRNKVRYAAIEITNTAQRLHQLNLTRFLPTAGVTVLLPAVIIHLLDVKSNDANVRMVSLQRFYQCMRILQRLREIYASADFATSFLEAAIRKAGISLNLSDLDEHEPVHPPPPAQTVRPAALTPPPESSAQKLPDLTYPSTFDTPSAGFTIPAEPSHNTTINPFDVATPPHSNSSENGSTQNLNPNLFKQQPDMSLNDFIDLANDAEVTQNDFDALLNFDDGGPELFGAEDGIMAFTFNSNHNSNNHQHDGHQSQRQPQTQGKSGFDYIPDFANDSNVNTLFSYDYSKRNDGFSGDVDPDMGLTLKL*

>jgi|PenchWisc1_1|145928|FarB|845aa

MTDTESGKRKASTAGLSGHNRPVKRRASKACCCCRARKVRCDVVENGSPCTNCRLDQVECVVTESKRRKKSRVEIENHSESPEALEENPSHRFSDLSGLADFAPTSPSQASVDLDQGQHMPHLLYQNQAQRIGSDDHYRRRMAPNPAVPASMPLANVTSQIQQLLDPNFGNTRSASGFLPDYIRGLPARLQKEDIDYLAVKGALTIPDVTLRNELLKSYIHYVHTYMPLLDLEEFLQNIVQNDGIHRISLLLFQAVMFAGVAFIDMKHLQAAGYQTRKAARKVFFQRARLLYDFDYEVDRISLVQSLLLMTYWYETPDDQKDTWHWMGVSLSLAHTIGLHRDPGNSRMDVCRQRMWKRIWWSTYTRDRLIALGMRRPMRVKDDDCDVPMLSLDDFEFHPFSPEIVAIVGNSEILQNVSHQRELALMFIEKAKLCLCVSHVLSAQYSVLSHKFGGTMETTMMLVPKKSTAETFEVRSCDQELEDWLANLPVETQYTASGGSKLSEAEEVLHSHRALLKMVYLTTSSALHRPQVLPAIPYPSTDAELQDMSRNKVRFAAVEITAIAQDLHCLDLTRYYPTTGVTVLLPAVIIHLLDIKSTDPSIRMTSLQRFYQCMRILQRLREIYASADFATSFLEAAIRKAGIQLTVSPQDVQSSSASSHTRDQPAASSDPFHSTATVPSRLHTLTPPPDSLAQKIPDLTYPKLSGPSAGGRTTGDLFASTPPHSDGSENGSTNNLNPNANHNKDRDAFAMPSEMSLTELMDLANDAEVTQNDFDALINFDDPGNTDFFADDNAQQNGAPVQEKGFDFEVDNVNMMGFDTDKASDFANLADNSMSEHEPMKLSG*

>jgi|Aspni_1|NRRL3_00665|FarA|912aa

MSTTENHSDLTASKTSPAPSTAGSTGTSGITVRTGPNGHMSFRRQRASRACETCHARKVRCDAASLGVPCTNCVAFSIECKIPTPKRKKNQTKSKETSGGEEAESKSPQSKPEPLPVESGKDAFGYQRNRMAVDGMPVTSLTESQAAQQASQNSMYAQFMKPKFARAPIKEAGRVAYLGESSNLSLLVQDRHGTTDVVHYPLPPNMGGTPARLTDLDSLELDILHQRGAFLLPPKPLCDELVEAYFKWVAPVVPIVNRSRFMRQYRDPKNPPSLLLLQAILLAGSRVCTNPQLMDANGSTTPAAMTFYKRAKALYDANYEDDRVTIVQALVLLGWYWEGPEDVTKNVFYWTRVAMVVAQGSGMHRSVESSQLTKPDKRLWKRIWWTLFTRDRSVAVALGRPIGINTDDSDVGMLTEDDFIEDEIDIAAEYPPDPVHVQFFLQYVKLCEIMGLVLSQQYSVASKSRRMNAMDLTHSDMALADWLQNCPKEVCWQRQRHHFWAALLHANYYTTLCLLHRAHMPPASSVSSNYRVEEMAYPSRTIAFQAAGMITSIVENLQNHTEIRYAPAFIVYSLFSALIMHVYQMRSSVPSIVATCQERINICMQALKDVSKVWLVAKMVHTLFESILGNKVLEERLQKAAGKRHQRVRPEAAQQPPAKKPEPPKRKFDEMEFGLPNGGGPTPPVSYERSRPQTPAVTPSREMQPPGLNLPQGSPPAPGQPGASRGNTRPTTPFNQYSLPATPPDLFLVTRTSPNLSPSLWENFQPDQLFPDGTTFFPELTSPQPNTVDPQLQMQSQLQAQGMDQRAMMPQQVPGRGSISAAQGSPEVMSTLTPAIGLPGQHSQQVFGIESQQGWPMQNMDATLGGAPMDAASQDDNWSSSSRSGPTAPTTLNVEDWFQFFGINGSFGDLAT

>jgi|Aspnid1|3134|AN7050*|FarA|923aa

MSAAAGDKFIDSSASRPSPTPSTAGSTGTAGISVRAGANGQMSFRRQRASRACETCHARKVRCDAASLGV

PCTNCVAFSIECRIPTPKRKKSQAKPREVGDSNGDGDDKSQSQEKREESLPMPGKDAFGYQNSNTSNTNA

MAVNGMPVTTLTEAQAAQQASQNSTYAQFMKPKFARAPIKEAGRVAYLGESSNLSLLVQDRHGTTDVVHY

PLPPNIRGSRARVSDLDNLELDILHQRGAFLLPPKSLCDELVDAYFKWVAPVVPIVNRSRFMRQYRDPKN

PPSLLLLQAILLAGSRVCTNPQLMDANGSTTPAAMTFYKRAKALYDANYEDDRVTIVQALVLLGWYWEGP

EDVTKNVFYWTRVAIIVAQGSGMHRSVESSQLSKPDKRLWKRIWWTLFTRDRSV

AVALGRPICINTDDADVEMLTEEDFVEDEIDIAAEYPPDPVHVQFFLQYVKLCEIMGLVLSQQYSVASKS

RRMNAMDLTHSDMALADWLQNCPREVCWQRQRHHFWAALLHANYYTTLCLLHRAHMPPASSVPSSYRVEE

MAYPSRTIAFQAAGIITSIVENLQTHNEIRYTPAFIVYSLFSALIMHVYQMRSSVPSIVATCQERINICM

QALKDVSKVWLVAKMVHTLFESILGNKLLEERLQKAAGKRHQRVKPDSNHSNQHLPSRRPDPPPKRKFDD

MDLALPNGGPTPPVSYERSRPQTPAATPSRELPQSTMSIPQTSPTAAKDGLPGAGNSRANTRPTTPFNAQ

FSLPATPPDLFLVTRTSPNLSPSLWENFQPDQLFPDGTAIFPELTSPQNTTVDPQLQMQSQLHTHDMVQQ

QMPPRTSLAGTQGSPEILSSMPPAIGMQGQPQQMYGMDPQQSWQMPGLDPTVAGAMDNASQDDNWSSSSR

SGPTAPTTLNVEDWFQFFGINGSFGEMAV

>jgi|Aspca4|1139506|FarA|913aa

MSSAENHTDPTASKTSPAPSTAGSTGTSGITVRTGSNGHMSFRRQRASRACETCHARKVRCDAASLGVPCTNCVAFSIECRIPTPKRKKNQTKSKDNTSGGEEVEEKSPPSKPEPPAIEAGKDAFGYQSNRMAVDGMPVTTLTETQAAQQASHNSMYAQFMKPKFARAPIKEPGRVAYLGESSNLSLLVQDRHGTTDVVHYPLPPNMGGTPARLTDLDSLELDILHQRGAFLLPPKPLCDELVEAYFKWVAPVVPIVNRSRFLRQYRDPKNPPSLLLLQAILLAGSRVCTNQQLMDANGSTTPAAMTFYKRAKALYDANYEDDRVTIVQALVLLGWYWEGPEDVTKNVFYWTRVAMVVAQGSGMHRSVESSQLSKPDKRLWKRIWWTLFTRDRSVAVALGRPIGINTDDSDVGMLTEDDFVEDEIDMVAEYPPDPVHVQFFLQYVKLCEIMGLVLSQQYSVASKSRRMNAMDLTHSDMALADWLQNCPKEVCWQRQRHHFWAALLHANYYTTLCLLHRAHMPPASSVSSNYRVEEMAYPSRTIAFQAAGMITSIVENLQNHQEIRYAPAFIVYSLFSALIMHVYQMRSSVPSIVTTCQERINICMQALKDVSKVWLVAKMVHTLFESILGNKVLEERLQKAAGRRHQRVRPETMQQPPAKRPEPPKRKFDEMEFGLPNGGGPTPPVSYERSRPQTPAVTPSREMQPPGLSLPQGSPPAPGGPGVSRGNTRPTTPFNQFSLPATPPDLFLVTRTSPNLSPSLWENFQPDQLFPDGTTFFPELTSPQPNAVDPQLQMQSQLQTQNLDQRPMIPQQMAARGSLSAAQGSPEVMSTLTPGVGLPGQQPQQVFGLENQQGWPMQSLDAALGGATMDAASQDDNWSSSSRSGPTAPTTLNVEDWFQFFGINGSFGDLAT*

>jgi|Aspfl2_32|63141|FarA|909aa

MSTTGENHTDSTSRPSPAPSATGSTGTSGITVRAGSNGQMSFRRQRASRACETCHARKVRCDAASLGVPCTNCVAFSIECKIPTPKRKKNQTKAKESSGSEENPQKETPKDDQSTTDGKDAFGYSSNRMAVDGMPVTSLTESQAAQQATQNGAYAQFMKPKFARAPIKEAGRVAYLGESSNLSLLVQDRHGTTDVVHYPLPPNIRGSRARLADLDNLELDILHQRGAFLLPPKPLCDELVDAYFKWVAPVVPIVNRSRFMRHYRDPKNPPSLLLLQAILLAGSRVCTNPQLMDANGSTTPAAMTFYKRAKALYDANYEDDRVTIVQALVLLGWYWEGPEDVTKNVFYWTRVAMVVAQGSGMHRSVESSQLSKPDKRLWKRIWWTLFTRDRSVAVALGRPIGINTDDSDVGMLTEDDFIEDEIDIAAEYPPDPVHVQFFLQYVKLCEIMGLVLAQQYSVASKSRRMNAMDLTHSDMALADWLQNCPKEVCWQRQNHHFWAALLHANYYTTLCLLHRAHMPPASSAPNSYRVEEMAYPSRTIAFQAAGMITSIVENLQTHQEIRYTPAFIVYSLFSALIMHVYQMRSSVPTVVATCQERINICMQALKDVSKVWLVAKMVRTLFESILGNKVLEERLQKAAGKRHQRIRHDTAQHQPPRKPDPPKRKFDDMDLGLPNGGPTPPVSYERSRPQTPAVTPSREMGQPGLNVPQGSPTGPPAGNSRGNTRPTTPFNAQFSLPATPPDLFLVTRTSPNLSPSLWENFQPDQLFPDGTAIFPELTSPQQTAVDPQLQMSSQLQTQGMDQRHMMPHQMSSRGLPGTQGSPEMISNIPPGLGMQGQQPPQVFGMENQQPWPMAGLEAALHTGVEAASQDDTWSNSSRSGPTAPTTLNVEDWFQFFGINGSFGDLSTSA*

>jgi|Aspte|5806*|FarA|912aa

MSTTGENHTDSTSRPSPAPSATGSTGTSGITVRAGPNGHMSFRRQRASRACETCHARKVR

CDAASLGVPCTNCVAFSIECKIPTPKRKKNQNKSKETSGSEAGSERPPTKQEEEAPAADG

KNAFGYRSNRMAVDGMPVTTLSDQQVAEQASQNNTYAQFMKPKFARAPIKEAGEGRLPGR

VVESVAPGAGSPWNDRCRALPPFHIRGSRSRLTDLDNLELDILHQRGAFLLPPKPLCDEL

VEAYFKWVAPVVPIVNRSRFMRHYRDPKNPPSLLLLQAILLAGSRVCTNPQLMDANGSTT

PAAMTFYKRAKALYDANYEDDRVTIVQALVLLGWYWEGPEDVTKNVFYWTRVAMVVAQGS

GMHRSVESSQLSKPDKRLWKRIWWTLFTRDRSVAVALGRPIGINTDDSDVGMLTEDDFIE

DEIDIAAEYPPDPVHVQFFLQYVKLCEIMGLVLSQQYSVASKSRRMNAMDLTHSDMALAD

WLQNCPKEVCWQRSRHHFWAALLHANYYTTLCLLHRAHMPPASSVPSSYRVEEMAYPSRT

IAFQAAGMITAIVENLQTHQEIRYTPAFIVYSLFSALIMHVYQMRSSVPTIVATCQERIN

ICMQALKDVSKVWLVAKMVHTLFESILGNKVLEERLQKAAGRRHQKVRPEANQQQALRRP

DPPPKRKFDDMDIGLPNGGPTPPVSYERSRPQTPAVTPSRELSQPGLGVPQASPTAHRDG

GPGNSRANTRPTTPFNGQFSLPATPPDLFLVTRTSPNLSPSLWENFQPDQLFPDGTAIFP

ELTSPQHNAVDPQLQMQTQLQTQGIDQRMMGQPVPSRSMSGTQGSPELLSSMPPGIPLQG

QQPPQMFGMEHQGSWPMQGLEATLSAATVDPANQDDNWSSSSRSGPTAPTTLNVEDWFQF

FGINGSLGEMAT

>jgi|Aspfu1|4956*|FarA|909aa

MSTTAESRPESTPSRPSPAPSTAGSTGTSGITVRGGPNGHMSFRRQRASRACETCHARKV

RCDAASLGVPCTNCVAFSIECKIPTPKRKKQQSKSKDTNSGTDERNEDGKDKQEESPGDG

TDSKDAFGYRKNRMAVDGMPVTTLTEQQEAQQASHNTAYAQFMKPKFARAPIKEAGRVAY

LGESSNLSLLVQDRHGTTDVVHYPLPPNIRGSRARLTDLDNLELDILHQRGAFLLPPKPL

CDELVEAYFRWVAPIVPIVNRSRFMRQYRDPKNPPSLLLLQAILLAGSRVCTNPQLMDAN

GSTTPAAMTFYKRAKALYDANYEDDRVTIVQALVLLGWYWEGPEDVTKNVFYWTRVAMVV

AQGSGMHRSVETSQLSKPDKRLWKRIWWTLFTRDRSVAVALGRPIGINTDDSDVGMLTED

DFIEDEIDSVAEYPPDPVHVQFFLQYVKLCEIMGLVLSQQYSVASKSRRMNAMDLTHSDM

ALADWLQNCPKEVCWQKSRHHFLAALLHANYYTTLCLLHRAHMPPASSAPNSYRVEEMAY

PSRTIAFQAAGMITSIVENLQTHQEIRYAPAFIVYSLFSALIMHVYQMRSSVPSIVATCQ

ERINICMQALKDVSKVWLVAKMVHTLFESILGNKVLEERLQKAAGKRHQRVRPETQTQQP

PAKKPDPPKRKFDDMDLGLPNGGPTPPVSYERSRPQTPAVTPSRELGQPPGISVPQGSPT

GPQNAMPGNSRSNTRPTTPFNGHFSLPATPPDLFLVTRTSPNLSPSLWENFQPDQLFPDG

TAIFPELASPQNHTVDPQLQMQSQLQSQGMQQRHMMVNQTPQMPGRHLSSPELMSGLPPG

MGLHGGQPQQVFGLENQHGWPMQGLEAALNATGMDNTSQDDNWSSSSRSGPTAPTTLNVE

DWYVCIRDA

>jgi|Aspor1_10271*|FarA|908aa

MSTTGENHTDSTSRPSPAPSATGSTGTSGITVRAGSNGQMGFRRQRASRACETCHARKVRCDAASLGVPCTNCVAFSIECKIPTPKRKKNQTKAKESSGEENPQKETPKDDQSTTDGKDAFGYSSNRMAVDGMPVTSLTESQAAQQATQNGAYAQFMKPKFARAPIKEAGRVAYLGESSNLSLLVQDRHGTTDVVHYPLPPNIRGSRARLADLDNLELDILHQRGAFLLPPKPLCDELVDAYFKWVAPVVPIVNRSRFMRHYRDPKNPPSLLLLQVILLAGSRVCTNPQLMDANGSTTPAAMTFYKRAKALYDANYEDDRVTIVQALVLLGWYWEGPEDVTKNVFYWTRVAMVVAQGSGMHRSVESSQLSKPDKRLWKRIWWTLFTRDRSVAVALGRPIGINTDDSDVGMLTEDDFIEDEIDIAAEYPPDPVHVQFFLQYVKLCEIMGLVLAQQYSVASKSRRMNAMDLTHSDMALADWLQNCPKEVCWQRQNHHFWAALLHANYYTTLCLLHRAHMPPASSAPNSYRVEEMAYPSRTIAFQAAGMITSIVENLQTHQEIRYTPAFIVYSLFSALIMHVYQMRSSVPTVVATCQERINICMQALKDVSKVWLVAKMVRTLFESILGNKVLEERLQKAAGKRHQRIRHDTAQHQPPRKPDPPKRKFDDMDLGLPNGGPTPPVSYERSRPQTPAVTPSREMGQPGLNVPQGSPTGPPAGNSRGNTRPTTPFNAQFSLPATPPDLFLVTRTSPNLSPSLWENFQPDQLFPDGTAIFPELTSPQQTAVDPQLQMSSQLQTQGMDQRHMMPHQMSSRGLPGTQGSPEMISNIPPGLGMQGQQPPQVFGMENQQPWPMAGLEAALHTGVEAASQDDTWSNSSRSGPTAPTTLNVEDWFQFFGINGSFGDLSTSA

>jgi|Aspgla1|46697|FarA|929aa

MNTAADSYHDSAAPAASSKVSPAPSTAGSTGTSGITVRTGPNGQMSFRRQRASRACETCHARKVRCDAASLGVPCTNCVAFSIECKIPTPKRKKNAKSKEPNGYETGNDERNDIKPRQTVFPSQEKDAAFGYRKDRMAVDGMPATTLTDSQAEQQDSQNSAYAQLMKPKFARAPIKEAGRVAYLGESSNLSLLVQDRHGTTDVVHYPLPPNVRGSRSTLTELDSLELDILHQRGAFLLPPKPLCDELVDAYFRWVAPVVPIINRNRFMCQYRDPKNPPSLLLLQAILLAGSRVCTNSQLMDSNGSTTPAAMTFYKRAKALYDANYEDDRVTIVQALVLLGWYWEGPEDVTKNVFYWTRVAMVVAQGSGMHRSVESSQLSKPDKRLWKRIWWTLFTRDRSVAVALGRPIGINTDDSDVGMLTEDDFVEDEIGLPSEYGPDPVHVQFFLQYVKLCEIMGLVLSQQYSVASKSRRMNAMDLTHSDMALADWLQNCPKEVCWQRSRHHFWAALLHANYYTTLCLLHRAHMPPASSVPSSYRVEEMAYPSRTIAFQAAGMITSIVENLQTHDEIKYTPAFIVYSLFSALIMHVYQMRSSVPSIVTTCQERINICMQALKDVSKVWLVAKMVHTLFESILGNKVLEERLQKAAGKRHQRVRPEGQHQYPPPPLRRPDLPPKRKFDDMDLGLPNGGPTPPVSYERSRPQTPAATPSRDDLPPPSASHLPQGSPGVPRESLTGTGLSRGNTRPTTPFNGQFSLPATPPDLFLVTRTSPNLSPSLWENFQPEQLFPDGTAFFPELASPHDGAVDPQLQMQSHLQTQNMQQRMMMNNQHMPRTVSGAGTQGSPEMLSAMPAGLSVQPQQSQQMFGVENQQTWPMQGLDPPLSTAGLENASQDDNWSSSSRSGPTAPTTLNVEDWFQFFGINGSFGEMAG*

>jgi|Aspcl1|1904|FarA|917aa

MSTIAENHSESTPSKPSPAPSTAGSTGTSGITVRGASNGHMSFRRQRASRACEVRCDAASLGVPCTNCVAFSIECKIPTPKRKKNQTKSKDTNSGNDEQNEDSQEKQETSPSDVPDGKNAFGYGKNRMAVDGMPVTTLSESQAAQQASQNNAYAQFMKPKFARAPIKEAGRVAYLGESSNLSLLVQDRHGTTDVVHYPLPPNIRGSRARLTDLDNLELDILHQRGAFLLPPKPLCDELVEAYFKWVAPVVPIVNRSRFMRHYRDPKNPPSLLLLQAILLAGSRVCTNPQLMDANGSTTPAAMTFYKRAKALYDANYEDDRVTIVQALVLLGWYWEGPEDVTKNVFYWTRVAMVVAQGSGMHRSVESSQLTKPDKRLWKRIWWTLFTRDRSVAVALGRPIGINTDDSDVGMLTEDDFIEDEIDIAAEYPPDPVHVQFFLQYVKLCEIMGLVLSQQYSVASKSRRMNAMDLTHSDMALADWLQNCPKEVCWQRSRHHFLAALLHANYYTTLCLLHRAHMPPASSAPNSYRVEEMAYPSRTIAFQAAGMITSIVENLQTHQEIRYTPAFIVYSLFSALIMHVYQMRSSVPSIVATCQERINICMQALKDVSKVWLVAKMVHTLFESILGNKVLEERLQKAAGKRHQRVRPEATHQHPAKKPEPPKRKFDDMDIGFPNGGPTPPVSYERSRPQTPAVTPSRELGQPPGISVPQGSPSAPHSTIPGNSRSNTRPTTPFNGHFSLPATPPDLFLVTRTSPNLSPSLWENFQPEQLFPDGTAIFPELTSPHTQTVDPQLQMQPQMQPQGLPQRPMMMNQATHMPGRSMSISGAQGSPELLSGMPPGMGMQGVQPQQMFGLENQHGWPMQGLDAPMSATNVDHASQDDNWSSSSRSGPTAPTTLNVEDWFQFFGINGSFGEMIT*

>jgi|Aspzo|1965052|FarA|995aa

MSTVADPSPSASSAGSVSHLANNNSALPAGPNSAQMSFRRQRASRACETCHARKVRCDAA

SLGVPCTNCVAFSIECRIPTPKRKKQQQQQQQPQQQEQHQTGSKEDSVSDDVTDSKFSGN

ETEKREPSPNEGNQGVFGYRNKRMPAEAMPNTALPESQPVPKEPYQASASAASSSPAPFM

KPKFSRAPIKEPGRVAYMGESTNLSLLVQDRHGSDDVVHYPLPVNIRGSRARLTDLDNLE

IEILHQRGAFLLPPKPLCDELVDAYFQWVAPVVPIINRSRFMRQYRDPKNPPSLLLLQAI

LLAGSRVCTNPQLMDANGSMTPAAMTFYKRAKALYDANYEDDRVTIVQALVLLGWYWEGP

EDVTKNVFYWTRVAIIVAQGSGMHRSVETSQLSKPDKKLWKRIWWTLFTRDRSVAVALGR

PIGINTDDSDVGMLTEDDFIEDEVDTVAEYAPDPVHVQFFLQYVKLCEIMGLVLSQQYSV

ASKARRTNAMDLTHSDMALADWLQNCPKEVCWQRSRHHFWAALLHANYYTTLCLLHRAHM

PPASSVASTYRVEEMAYPSRTIAFQAAGMITSIMENLSAHHEIRYTPAFIVYSLFSALIM

HVYQMRSSVPSVVATCQERINICMQSLKDVSKVWLMAKMVHTLFESILGNKALEERLQKA

AGRRHQKTRPDSFGGNGFNNANTTTTTNNNNGHYNYSGHRSATPVPSRRTDPPKRKFDDM

DLGLPTGGPTPSVSYERSRPQTPAVTPSRDLGQAPMSPNAPRAQTDALPGTAAAVAAGPG

GTGNSRSNTRPTTPFNGQYSLPATPPDLFLVTRTSPNLSPSLWENFQPEQLFPDGTSIFP

ELASPQTSTVDPQLHMSTHMPAQNIPSRPVMYGQQQQQPPPPPPLQQSATAHQMTLGQGS

PEFLSTIPPEMGLQPPPPHPAAAAVQPVFGLESQPWAMQGLDAPLSGAPLDAASQDDTWS

SSSRSGPTAPTTLNMEDWFQFFGINGGFPDLSASH

>jgi|Talst1_2|12430|FarA|941aa

MVAENQMAGQTEQQEHQEQQQHPSPASSQDRSKLGSNNQSQSTPSSSTVPNAGIASFRRQRASRACEVRC

DAASLGVPCTNCVAFAIECRIPTPKRKKNQQPRNKDNNGDTKNHDQSKDDDDDEDPNTPRTHENPVFSAN

PPISIDGMPANTLATASQPNPEKHNPGHYSAQIMKPKFTRAPIKEPGRVAYLGESSNLSLLVHDRGHTDV

VHYPLPETIRGSRGHLTELDSLEIEILHRRGAFLLPPRDLCDELVEAYFKWVAPVVPIVNRNRFMRRYRD

PQNPPSLLLLQAILLAGSRVCNNSKLMDQNGSTIPASTTFYKRAKALYDANYEDDRVTLVQALVLMGWYW

EGPEDVTKNVFYWSRVAILVAQGSGMHRSVENSQLSRSDKRLWKRIWWTLFTRDRSVAVALGRPININID

DSDVEMLTEDDFIEDEDDIPAEYPPDPIHVQFFLQYVKLCEIMGLVLSQQYSVASKSRRTNAIDLTHSDM

ALADWLQNCPRAVYWERKNHHFWAALLHSNYYTTLCLLHRAHMPPATASANSYRTEEIAYPSRTIAFQAA

GMITSIVEKLQSHNEIRYTPAFIVYSLFSALIMHVYQMRSSVPSVVAACQERMTICMQALKDVSKVWLVA

KMVHTLFESILGNKVLEERLQKAPGKRHQKNRPSEHEQNIASNHKKTELPKRKFDDMDLGLPNGNPTPPV

SYERSRPQTPAATPSRELNSMTGQQGGMGSLPQMSPNLLKGQDGAQNTGMSRAQTPFNPSYSLPATPPDL

FLVTRTSPNLSPTLWENFQPDQLFPDGTNFFPTPANMMDPQMQSTTNTMGGQGNMMMNHHQQQQQQQMPS

RTLPGAQGSPSLVSSGGIHPDMGNLPPQPSQQMFGMEGMQNWAGLDAALAGNPDSTSQDDNWSTSSRGNP

TAPTTLNVEDWFQFFGINGGFGDLITDPMTG

>XP_002561763.1|Pc16g14660|[Prubens Wisconsin 54-1255|FarA|930aa

MSTTEFHTDQLSDNHTSPAPSNAGSTGTSGITVRNGPQGQPLSFRRQRASRACEVSLLLVVVFFPSNRSC

RLVRCDAASLGVPCTNCVAFSIECKIPSPKRKKNNQNKNKDDTREKKDKNSDSDEKSNAFGYRNNLMGVD

GMPNTSLSEAEAAQQASANNAYAQFMKPKFARAPIKEAGRVAYLGESSNLSLLVQDRHGTADVVHYPLPP

NIRGSRARLTDLDNLEIDILHQRGAFLLPPKPLCDELVDAYFKWVAPVVPIINKSRFMRHYRDPKNPPSL

LLLQAILLAGSRVCTNQQLMDANGSTTPAAMTFYKRAKALYDANYEDDRVTIVQALVLLGWYWEGPEDVT

KNVFYWTRVAMVVAQGSGMHRSVEMSQLNKPDKRLWKRIWWTLFTRDRSVAVALGRPIGINTDDSDVEMV

TEDDFIEDELDIVAEYPADPVHVQFFLQYVKLCEIMGLVLSQQYSVASKSRRMNAMDLTHSDMALADWLQ

NCPKEVCWQRSRHHFWAALLHSNYYTTLCLLHRAHMPPASSAPNNYRVEEMAYPSRTIAFQAAGMITSIV

ENLQAHGEIRYTPAFIVYSLFSALIMHVYQMRSSVPSIVATCQERINVCMLALKDVSKVWLVAKMVNTLF

ESILGNKVLEERLQKAAGRRHQRTRHGESSSSKRHDPPKRKFDDMDIGMPNGGGPTPPVSYERSRPQTPA

VTPSRELNQPLPGQQSPNAHRGPHDPMTGTGNSRANTRPTTPFNGQFSLPATPPDFFLVTRTSPNLSPSL

WENFQPDQLFPDGTAFFPELTSPPQPGAVDPSLQIPSQMPAGMASRPSPMMSNQPQPVSGRSMSISHGSP

PIMSGLPGAMGMHPNPPQQMFGMEGQHQQAWPPMHGLDGTMSGAAMDAASQDNDTWSSSSRSGPTAPTTL

NVEDWFQFFGINGGFGDLAA

>jgi|Aspni_NRRL3_1|1945|FarD|817aa

MLTAKRKPSSADLGDDRPVAKRSNSLSHNAHAPWLQESRPSRSWRTLGDRGVRTSLPLENMVSTVRDLIDPDFDPLIALLDDEPRFLKPLPSRIPPEDLEFLRFRGALSIPESGLRNELLRCYIKWVHSFMPVLNLQEFLRCVAENDPNGNISLLLFQAVMFVATAFIDLHHLQAAGYATRKIARNVFFTRLRLLYSLDCEDDRIVILQTLLLMTYWSDHQNNPQRDIWDWIGVCNIHAHSIGLNRDPSSSTNMDPRTKRLRTRIWWSLYSRDRLIAMGLRRPTQVNEGTSNVPMLKLEDFDFESFHPSVIEMFRCRQLEDVTHQKRLATMFIEKAKLCQCIGRVLFAQYTPSQCQFGVTTRTTISLVPRQASESELARCSQRLESWLSALPKDAQFIPASRTNFHDGEDVLLLHGAMVRMLYHATTSALHRPWAFTPNKDQSKSRRELIQAAQTKMHDAAVGITHIIQGLNQLNLTRFLPQSGVTVIIPAAVAHLTNSSSGNPATRESSIYNFQRCIQVLQGLRDIYPAADMEVANIEAAVKLQSDSANTFLRIMQYTNNSGSMSQGQSQEHARKQSTISNPHTIASAEDRTSNHWTPPQDGPDPTNNHKQSEPQHPAGPTKQQQQRTPTKRHSSTINPPLIFNNTSTTNNTNNPSNPLPKKAEPTPPNDFDDDPISNFLNDNNPTISPRNTQTQPNPTFDQGSDQFPFDFDLDLDSFAADFPPQNPPSTNLNPASTSNPDASPDLDIDWASELLRWTGPGPEDALTNNNDLFSPFAPTPNVESTPGQRSMSHVTGDITGDLDRDLGFVPSDEEIF*

>jgi|Aspca4|1142122|FarD|803aa

MLTAKRKPPNADLNDDRPVAKRSNSLSYNAHAPWLQDSTPSRTWRTLGDRGVRSSLPLENMVSTVRDLIDPDFDPLIAILDDEPRFLKPLPSRIPPEDLEFLRFRGALSIPESGLRNELLRCYIKWVHSFMPVLNLQEFLRCVAENDPNGNISLLLFQAVMFVATAFVDLQHLQAAGYATRKTARSAFFTRLRLLYSLDCEDDRIVVLQTLLLMTYWSDHQNHPQRDIWDWIGVCNIHAHSIGLNRDPSSANMDPRTKRLRARIWWSLYSRDRLIAMGLRRPTQVNEGTSNVPMLRLEDFDFEPYHHTVIDMFRCRQLEDVSHQKRLATMFIEKAKLCQCIGRVLFAQYTPSQCQFGVTTRTTISLVPRQASESELARCSQRLDSWLSALPKDAQFIPASRTNFHDGEDVLLLHGAMVRMLYHATTSALHRPWAFATNKDLSKSRQDLIQTAQSKMHDAAVGITHIIQGLNQLNLTRFLPQSGVTVIIPAAVAHLTNSLSGNPATRETSIYNFQRCVQVLQGLKDIYPAADMEVANIEAAVKLQSDSTNTFLRIMQYANTSGTVTQLQSHEQPRKQSIISNPHTITSTDDRVSNQWTPPQDEPEPTTNPKQPTPKPTSPTTQPTPTKTTTTTNSLPPTFNNNHNCNSASNSTITPLPRLTEPTPPNDFDDPIANFLNDTNPPISPRNTRSQSNPNNTSTVNPPDPFPFDLDFDLDTFAADFPPQNPNPDPTADLDIDWASELLRWTGPAPEDALTNNKDLFSPFPNHIHGSTPGQGSSSHATGDITGDLDRDLGFVPSDDDIF*

>jgi|Aspfl2_3|8529|FarD|765aa

MITTKRTASDADIHYGEPVAKRNSLGYDARTPWLQDSKPPPTWRTLGNRAVRPSMPLENMVAAVQDLIDPDFDPLIAILDEEPRFLKPLPSHISPEDLEFLRFRGALSIPESGLRNELLRCYIKWVHSFMPVLNLQDFLRCVAENDPNGNISLLLFQAVMFVATAFVDLKHLQDAGYATRKIARSAFYTRLRLLYSLDCEEDRIAILQTLLLMTYWTDHVNHPQRDIWDWIGVCNTQAHSIGLNRDPTTSPTMDLKTKRLRIRLWWSLYSRDRLISLGMRRPTQVNEGTSNVPMLRLDDFEYDSFHPSVINMFHCRQLEDPSHQKRLATMFMEKTKLCQCIGRVLFAQYSPSQRLFGITNRTTITLAPRQASESELARCSQRLDSWVNSLPKDAQFIPASKTNFNDGEDVLLLHGAMVRMLYHATISALYRPWAYGSNKGQSKSRIELTNTARSKMHDAAIGITHIIQGLNQLNLTRFLPQSGVTVIIPATVLHLANSMSDNPTVREASIRNFHRCVQVLQGLKELYPAADMEVANIEAAVRVQSDSASTLLKIMQSNSINLSQPQPAEPYRRGSDVATVQTLSPTEDRTPKHWTSPIETDTVNESHNPGLEPRKPSSADQRSKRNSTVISTTVNTTSTSPSKAKQDYLTPTNDFDDHFNSAFNSHSPLPDPSFNPSSFLDIDSNNTEFPLFQSSSHPDIDIDWAEDFLRGADFKIDFSSSALEDHSRDFFSFSDKQERSNAPRKEEITGDLDKDLGLNSGDEMF*

>jgi|Aspte1|5380|FarD|772aa

MTTKRRSPNIEGRDNVPVARRDSVLGYNAHAPWLQDARPSKGWRTLGDRAVHASLPLANMVSTVQDLIDPDFDPLIAILDDEPRFLKPLPPRISPEDLEFLRFRGALTIPESGLRNELLRCYIKWVHSFMPVLNLQEFLRCVVENDPNGNISLLLFQAVMFVATAFVDLKHLQDAGYSTRKSARSAFYTRLRLLYSLDCEDDRIIILQTLLLMTYWTDHMNHPQRDIWDWIGVCNTVAHSIGLNRDPSTATNMDDKTKRLRIRLWWSLFSRDRLIAMGLRRPTQVNEGTSNVPMLKLEDFDPEPFHPAVIEMFDCRQLQDASHQKRLAIMFIEKTKLCQCIGRVLFAQYMPSQRLFGTTTRTTITLIPRQASESELARCSQRLDSWLGALPKDAQFIPASKTNIKEGEDVLLLHGAMVRMLYHATISALHRPWAMGANKDQPKSRNELTNAARAKMHDAAIGITHIIQGLNQLNLTRFLPQSGVTVIIPAAVAHLANSLSDNPSIREASIHNFQRCIQVLQGLQDIYPAADMEFANIEAAVRLQSDSASTFLRIMQYNTINMSQLQAQSQSRRPSAGSTVQTITSPDERTPSHWASPKAAEPNSSSRPHPPENKTPKPTSPETRKDSTATTAANEPSSKKAKLEHPTPSTSTDFDDPFSRMINPPPSPPADTNPTIFNPSDLLRMDIDSYISDFPLPDPSGYPNLDVDWAEELLRGAELDLDYPGPTTDDRSNDIFDFNDKPERGSMGHGNGDITGDLDRDLGLTHSGDEMF*

>jgi|Aspfu1|8695|FarD|738aa

MVTTKRKLSSVDIDAELPNTKKSNLLGYDARTPWLFDSKPSSLGRRTAGNPAVQHSLPLANMVSTVQDLIDPDFDPLIAILDDEPRFLKPLPSRISSEDLEFLRFRGALSIPESGLRNELLRCYIQWVHSFMPVLNLQEFLRCVAENDPEGNISLLLFQAVMFVATAFVDFKHLQDAGYKTRKSARNAFYERLRLLYSLDCEEDRIAILQTLLLMTYWSDHVNNPQRDIWDWIGICNTQAHSIGLNRDPTTSDMDPRMKRLRVRLWWSLYCRDRLIAMGLRRPTQINEGTSSVPMLRLDDFDFEPFHPSVIEIFRCRQLEDVSHQKRLATMFIEKAKLCQSIGRVLFAQYATSQCQFGVTNRTTITLVPRQASESELARCSQRLDSWLSALPKDAQYIPASKTNFEDGEDVLLLHGAMLRMLYHATISALYRPWAYSRDPDKSRFEITQNARSKMHDAALGITHIIQGLNQLNLTRFLPQSGVTVIIPAAVAHLSNIMSNNPAVRETSLQHFQRCIQVLRGLRDLYPAADMEVANIEAAVKVQSDSVSTFLKIMEYSNVSIDQLQPPEPPLHKQSIDSSMHTACSPEGSSPDRRGSFVGATDAQPKEHRTGSLQTNRASIATSISRSNNEPEHTNHQQQQPTPTNDFDDHLNNFPSPQTISSFIPADLLRIDTDSFPDFISPDPNISSVPELDVDWTDELLGGTELSSEYPETGSVSHSHGGITGDLDRDLGLALDSY*

>jgi|Aspor1|9360*|FarD|765aa

MITTKRTASDADIHYGEPVAKRNSLGYDARTPWLQDSKPPPTWRTLGNRAVRPSMPLENMVAAVQDLIDPDFDPLIAILDEEPRFLKPLPSHISPEDLEFLRFRGALSIPESGLRNELLRCYIKWVHSFMPVLNLQDFLRCVAENDPNGNISLLLFQAVMFVATAFVDLKHLQDAGYATRKIARSAFYTRLRLLYSLDCEEDRIAILQTLLLMTYWTDHVNHPQRDIWDWIGVCNTQAHSIGLNRDPTTSPTMDLKTKRLRIRLWWSLYSRDRLISLGMRRPTQVNEGTSNVPMLRLDDFEYESFHPSVINMFHCRQLEDPSHQKRLATMFMEKTKLCQCIGRVLFAQYSPSQRLFGITNRTTITLAPRQASESELARCSQRLDSWVNSLPKDAQFIPASKTNFNDGEDVLLLHGAMVRMLYHATISALYRPWAYGSNKGQSKSRIELTNTARSKMHDAAIGITHIIQGLNQLNLTRFLPQSGVTVIIPATVLHLANSMSDNPTVREASIRNFHRCVQVLQGLKELYPAADMEVANIEAAVRVQSDSASTLLKIMQSNSINLSQPQPAEPYRRGSDVATVQTLSPTEDRTPEHWISPIETDTVNESHNPGLEPRKPSSADQRSKRNSTVISTTVNTTSTSPSKAKQDYLTPTNDFDDHFNSAFNSHSPLPDPSFNPSSFLDIDSNNTEFPLFQSSSHPDIDIDWAEDFLRGADFKIDFSSSALEDHSRDFFSFSDKQERSNAPRKEEITGDLDKDLGLNSGDEMF*

>jgi|Aspgl1|82622|FarD|750aa

MLAAKRKPLDADLYHDDDGEQPPAKRNHSLDFNARAPWLQSSRPEREWRTMGNRAVQPSLPLANMVTAVQDLIDPEFDPLIAILDDEPRFLKPLPSRMVPEDLEFLRIRGALSIPESGLRNELLRCYIKWVHSFMPVLNLQKFLFSIAQNDPNGNISLLLFQAVMFVATAFIDFKHLHDAGYTTRKSARNAFYTRLRLLYSLDCEEDRIAIIQTLLLMTYWSDHANHPRRDIWDWIGVCNTQAQSIGLNRDPTSAPNMDPSTKRLRTRLWWCLYSRDRLIAMGLRRPTQLNEGTSNVPLLKLDDFDFETYHPSVTAMFHCRQLEDVSHQKRLATMFIEKARLCQCIGRVLFAQYAPSQCQFGTTTRTTITLVPRLASESELARCSQKLESWLNGLPKDAQFIPESRNNIREGEDVLLLHSAMLKMLHHATISALYRPWATNFQKDQSKARQELMNTARAKMHNSALGITHIVQGLNQLDLTRFLPQSGVTVILPAAVAHLTNSKSDNPTVRETSLYNFQRCVQVLQGLKDIYPAADMEVANIEAAVKVQSDNANTFLRIMQYSSSNSDSGQKSDSLEENPENPPNKETGAQQQTQQHRKQSTVSDLQSTTATATKPSPSPTAPTPTPSQKDHNNSYTDFNDNLDHLNLNTPPTNFLSFDLPNPPFDFATPADLEQNVSPTDAEPYGIDWTQELLAGGIDSDYGGPGFENERRDAWPFSPENGSNGIGSGSGHAHMTPGDITGDLDRDLGL*

>jgi|Aspcl1|4330|FarD|761aa

MMTAKRKLSMADTDLDTPIAKKSNFMGYDARTPWLYDSRPTLNRRTSGNPAVQHSLPLANMVSTVQDLIDPDFDPLIAILDDEPRFLKPLPSRISPEDLELLRFRGALSIPERGLRNELLRCYIQWVHSFMPVLNLQEFLRCVAENDLNGNISLLLFQAVMFVATAFVDFKHLQDAGYSTRKSARNAFYERLRLLYSLDCEEDRIAILQTLLLMTYWSDQVNNPQRDIWDWVGICNTQAHSIGLNRDPTTSNMDPRMKRLRVRLWWSLYCRDRLIAMGMRRPTQVNEGTSSVPMLRLDDFDFEPYHPSVIEIFRCRQLEDTSHQKRLATMFIEKAKLCQSIGRVLFAQYTTSQCQFGVTNRTTITLVPRQASESELARCSQRLDSWLSALPKDAQFIPASKTNFKDGEDVLLLHGAMLRMLYHATISALHRPWAFHSNKDQAPSRLELAQTARSKMHDAALGITHIIQGLNQLNMTRFLPQSGVTVIIPAAVAHLSNMMSDNPIVRETSLQHFQRCIKVLQGLKDLYPAADVEHANIEAAVKVQSDSISTFLKIMQYSDMKASQLRGPEPPLRRGSTDSIIHTISTEDRSPEQRDSLLGGTPRQRTESDVHRKDGHTERTSTKKESIATANLHNGSQPTNQQQQQRPIASNDFDDHFSLFPSPGSTSPFITTDLLRIDMDSFPDFKPMDTNMSSTPELDVDWTDELLGGSDLHLDGSGSSVDEPKDFFSFPSRREPGSISHSHCGITGDLDRDLGLALDSY*

>jgi|Aspnid1|8546|FarD|788aa

MLNAKRKQSEAGLTDDGPARRRSSNILGYNAQTPWLHESHDSRFSAIRRSIGTRGHAASLPLANMVSTVQDLIDPDFDPLIAILDEEPRFLKPLPSRISAEDLEFLRFRGALAIPESGLRIELLRCYIKWVHSFLPVLNLQEFLRCVALNDPEGNISLLLFQAVMFVATAFVDLKHLQAAGYTTRKSARNAFYTRLRLLYSLDCEEDRLVIVQTLLLMTYWSDHMNNPQRDIWDWIGICSTNAHSIGLNRDPSSSDLDIRTKRLRTRLWWCLFSRDRLIAMGMRRPTQVNEGSSNLPMLRLDDFDFEPFHPAVIEQFQCRQLEDVSHQKRLATMFIEKAKLCQCIGRVLFAQYTPSQCQFGLTNRTTINLVPRHASESELARCSQRLESWLSALPRDAQFVPASKTIFNDGEDVLLLHGAMIRMLYHATVSALHRPWAYGSTKDQTKSRLELAAAARTKMQDAAIGITQIIQGLNQLDLTRYLPQSGVTVILPAAVAHLANSMSNDPTLRENSISNFQRCIRVLQGLKEIYPAADMEVANIEAAVKAQSNTSALLRIMQFNGSLPSRPGSPHRTSLVSNPARIPSPLDEPNHWTPPADEQDTAHPILHQLGHASPDNKDKRSSPLKPNHEHSAQSNPPRQDQPTPPTDIPDPVTISPTTPQQNQNQPPVNPPKSSIFTDLLDFDLDTYTSTFTPSNPNPAAGPDLDLDWTSELLRWADANPEYYSAPNTNNDQRDIFSFPVGPGPGHNPTDLGLGNDGGSRGHGNLSSEITGDLDRDLGFTGDGEELF*

>jgi|Aspzo|92402|FarD|694aa

MAVKRKVSHAFTETEGDSNRVRFETCRPSTAWNNRPSHPWQRTMTNATMGSAISDLEFDPLTAIWDKEPRFFKPLPSRIPSEDREFLRFRGALTTPESGLRDELLRCYIRWVHGSLPILDLHRFLSIIAQNDPHGKISLLLFQTVMFVGTAFVDIKHLQTAGFATRKLVQDEFYRRVKLLYTFECEEDRLALLQSYLLLTSCSDQENTPHTDTWDLVASCTTLAYSIGLDQDPSQTALDPGMKHLRVRLWWSLYTCDRMTAMELRRPPHINGTPSDAPILTLEDFDLKPFHSFVVERFQCRQLENVCDQRRLATMFIEKVKLCQHIGEILSIRYSPLHRDLGRTSQPTITLAPRQAPESEMTQCNRRLELWAKELPQDAHFISPSSPKPTMKDGDDVLFIHSSMLKMLWHAASSAFHRPWLANSRIANRVTAGQLRSRDSVMGIQEISQNIYRLNLIRFLPQCAVMIISLAAAVHLEDSMAENPAVRNSSMARLQACIQVLIQVKEIYPSAVVELARLEAAVRKQSACHDIYSPILLPELNNCSNTSPPQAPIHIPPDIHPNLQQANKQATRNMASELGFPRPTPPVSYNLDSIFQSPPSSTAIDLDTFPGLSPDESLIDSEIPDLEADWTADLVDEWAKPDFNRDYTFSSVSDQKRHDSFSLSTPIYKDLGRRHITGDLDTDLGLLPFGDEFF*

>jgi|Talst1_2|7404|FarD|701aa

MSPKRKFSDVDSSAMYSTGVISADDAPSHKSRSFGRNVDRRNSSWRVMGDNAVPSSLPLSNLTDAVRELIDPDFHPELEQFLFDDESPQFLKPVSPRIATEDVEYLRSKGALTIPEPSLRNELLKAYVQWVYSSLPVLDLHSFLASVAQNKPDANISLLLFQAIMFAGTAFVDIRHLQAAGFKTRREARKAFFNNTRLLYVFDYEDDRIAIIQSILLMTYYYEKEETFQKDIWHWIGVCYTQAQSIGLHRDPSKSSINEHTKRLRIRLWWCLYSRDRQIALALRRPTQINEGICNVPLITLTDFDIRPYGDAVDQILPECRHMSDTYLQRRLAIMFIETVKLCQCLGRALFAQYTPSNFVSDVTQETTITLVPRQASDAELQRCGQKLDAWLGNLPEEATCVTPRERKDLKPGDDVLFLHASMLQMIYHATCTVLYRPRASSSNITTTLTTSKTSISAAQKIIRDAALHIADTVQHLRTLGLTPYLPTYGLTVMIPAAVAHLTDLKSANPVVRDGSKWNFRLCIEIISGLRDIYPAADYETECLEKAFQLQHGQRMLKSVYVMQDATVHSPRGAPTFVNTTELDLSILRPPREPSQIIESPAWTFDWQQDQDMFHRDGSDKDNDCWTINLDNDLDIGHFGLLSDGLFNNTGDGVPPHILMIQGDNDDIELSQNPDLPLTISREGEETTITGDLERDLGFI*

>jgi|PenchWisc1_1|146067|FarD|763aa

MSSAPGLKRKLESQEPTAKRHHLHGYDPQSPWLHESEPARIWRTRGNPGVQHSLPLSNMVSAVQDLIDPDFDPLTAMLDDEPRFLKPLPARIAPEDLEFLRFRGALSLPESGLRDELLRCYIQWVHSFMPVLNLQEFLRCVAENDPEGNVSILLFQAVMFVGTAFVDLKHLQAAGYSTRKSARNAFFTRLRLLYSLDCEEDRIEILQTLLLMTYWSDSENSPQRDIWDWIGVCNTQAQSIGLNKDPASGEIDTRTRRLRSRLWWCLYSRDRLIAMGMRRPLQVNDGTSSVPMLKLDDFDFEPFSPYVVAKFYCRQLQDVSHQKRLATMFIEKVKLCQCIGRVLFAQYTPSQRQFGATNRTTVTLVPRQASESEFARCGQKLDSWLSAVPKDAQFVPKSGKNFQDGEDVLLLHGAMLRMLYHATSSALHRPRAIASKDQSKSRTDWRNAARTKMNDAAAGITHIIQGLNHLNLTRFLPQSGVTVILPAAVAHLTNTMSDDPAVRESSVDNFHRCIEVLHCLKDIYPAAGQEFANIEAAIKMQAGASSTFFQVMEYNLDAPATSLATRKPSNANSISQPQPNPPVTVTETPKARERTPSTQDNMHQRNSSKETTHEHPPPLFPNDFDHYTDLGDIDNNPFNFLSIDIDSFPDTQTPSNPMKPNNTDKPYISPPPQDQDTVDWAQELFQDTDLNQYSNTEPFREPEKTTPPLDQDQTNDDPFSFTLNDRDDVRPVHSRHQSINEARPEITGDLDRDLGFQSEDDIF*

>jgi|Aspfl2_3|2227896*|FarC|546aa

MAEYHSTCHPDHLEPLELEILHRRDAFRLPPKAVQDTLVEVFFKWVAPILPVVDRDAFLR

QYESAEDSPSILLLQAMLMVASRCSTSEQRSKEYTVSPRTFYKKAKALYDAGYETNLITV

VQAVVLLGAYWEGPDDLTESGIFYWSRLGIALAQELGLHDSERYTGLQPSERGLRKRIWW

TLYTRDRSVAAAFGRPLHINPNYCTVEPLTESDFVEYDGNAPSEPTGEVQARFFMEYVKL

CQLMDLGLCLNLSARSTQDARSAGAAQCELGLNEWLVACPPELHWRQTRHTFLSAILFST

FYTIVCQLHLLQAPFSSKESQSSAFHAASTIVSIMETLLSHNELQYSPTFIICHAVTSFV

TLKHQMDASLPSLLHGIRLTLESNLELLEALAKTWPIATLFLEFFQTMTAPDQFNKLLSV

AVEECHKRAIGDKQDDPEAPRRPTSFKRPKLQQVVLPQSRVVFQILARETQRRQTALLRS

HGSGTASREVETTSFGSGSGATPGSADDISPGDLGDALESCEPTAVLRNLREIIRIGNSQ

GADNAT

>jgi|aspgl1|141075*|FarC||545aa

MAQPSIPPHPADFDPLELEILNRREAFSLPPKDVCDILVKIFFEWIAPILPVINRQDFMR

KYHSLDETGPPILLLQAMFMVAARFTANQQSSDGRSISPRVFYKKTKALYNAGYEKDSVT

ILQAVILLGMYWDGPDDLTECGIFYWSRLGTALAQAYGIHDREIYTDLNHSEKSLRKRIW

WTLYTRDRSVAAAFGRPLHIDLSDSTVDPLTESDFIERDEQLQIEYPSDKTQARFFIEYV

KLCQLMDLGLCLKLSSRSIQDNRRAEAAQCELGLSEWLAFCPLELQWRQSRHTFLSGVLF

SAFYTIICQLQLLQEPDSSNESQKSSFHAASTVISILETLYSHGQLQYAPSFIICHAVVS

FVTLQHQMEASVPSLLHAIRLKLDANLEMLEVLSQTWPIAGMMLELFQTTATPAHFDRLL

TAVVDDCHRRSLGETNDTSSGSRSFQRSRLRQVILPQSRVVLQLLARTSQNRMTAISRPI

LGDVYSDAEVENESAGLRPGEIHLNGSPDAFSSALETDPSAILQNLQEIIRIGRSNMPNN

DHHLS

>jgi|aspcl1|6895*|FarC|545aa

MAQMASNTHPDHLDPLELEILNRRNAFDLPPRTVCDALVDVFFKWISPVLPVVNNHDFMR

KYNDPQNPPSILLLQAMLMVASRYYHDESSQGCGISPRICYKKVKALYDAGYESDSTAIL

QAVVLLGVFWDGPDELTENGIFYWSRLGIALAQEQGLHEGRETYALLSPENQSIWKRIWW

TLYTRDRSVAAAFGRPLHINAEDCTVETLTGKDFIEYDEQTGIRQPTDDTAAQFFIQYVK

LCQLMDLGLCLKLSSRSTQESRTRGAAQCELGLNEWLTDCPFELQWRQSRHGFWPAMLCS

TFYTIVCQLHLLKAPYSSQESQSSAFHAASTVISILETLQSRDELKYTPSFIICHAVVSF

VTLKCQMEATVPSLLHAIRLKLEANLDMLGALAPTWPIASMFVELFRSMTGPEQFSPLLS

VATDNCRKRARGEEDGGTVPSRSTGPFKRPKVQQVILPQTKVVLQILQREAQRRLPLSSS

LQNASHQESERKHLGANFDNKHSTPSDEVEPEPENYDASTILQSLRQVIEIAKSTSLHFQ

DGNVS

>jgi|aspte|8707*|FarC|538aa

MAHSLAAPHPVHLDPLELDILNRRDAFGLPGREICDALIEIFFSRVAPILPVVNRHDFMR

RYHDPANRPSILLLQAIFTVASRFLTDNLSSGNSANTPRAFYKKAKALYDAGYEQDNIAV

IQAVILLGLYWDGLDDLVENGIFYWSRLGTALAQAEGLHQSETYVDLDPSESSLRKRIWW

TLYTRDRSVAAAFGRPIHINTDDCTVGELTVTDFVEHDEYLQLEYPVDVTHARFFIEYVK

LCQLMDLGLCLKLSSRSTHDSRNAEAAQCELGLNEWLVSCPAELHWRQARHSFLPAVLFS

QFYTIVCQLQLLQEPCSSSESQRSAFNAASTVISILETLQSHGELRYAPSFTIWHAVVSF

VTLKNQMSASVPSFLHAIRLKLDANLEILQTLSKTWPIARFLVELFNTMSTPEQFSRLLT

AAVEECQKRARGDFDSPGTGPRSSGPFRRPKIQQVVLPQSRVVLQILARESQRRQSPLPQ

LPRMDTATGPSDCRPENIPQEGVDGLAATIEDCEPTAILRNLQKIIDVGKSAAHRADE

>jgi|Aspfu1|1526|FarC|503aa

MNARNDTREGEKERQRRRQRGLLFQNDFLGWDVRESPRSAPSITRSTRSCTEYGYTSRNPILVDHLDPLD

VDILHQRHAFDLPSQAVCDALVDVFFKWIAPVLPVINRHDFMRRYRNPQDPPSILLLQAVLMVASRFHYD

AQSSGNGIISPRILYKKVKALYDAGYERDPTTVLQAVVLLGVYWDGPDDLTESGIFYWSRLGIALAQENG

LHKSENYVTLSATKRRIWKRIWWTLYTRDRSVAAAFGRPLHINSEDCTVEDLKESDFIEYDEENDSATDR

TAIVFFMQYVKLCQLMDLGLCLKLSSRSTEDSRMKGAAQCELGLNDWLVSCPSELQWRQSRHNFWSAILY

STFYTIVCQLHLLQAPDSSKEAQSSAFHAATSIVSILETLQSRGELKYTPSFIICHAVVSFVTLRSQMEA

SIPSLLHAIRQNLEANLDMLQVLSHTWPIAAMFVELFRIMTGPEQFERKLSVAAEKCRKRSSFRFCKGKP

RSNLTRKAHSTTS
